# Accelerated flowering time reduces lifetime water use without penalizing reproductive performance in Arabidopsis

**DOI:** 10.1111/pce.13527

**Published:** 2019-03-12

**Authors:** John N. Ferguson, Rhonda C. Meyer, Kieron D. Edwards, Matt Humphry, Oliver Brendel, Ulrike Bechtold

**Affiliations:** ^1^ School of Biological Sciences University of Essex Colchester UK; ^2^ Institute for Genomic Biology University of Illinois at Urbana‐Champaign Urbana Illinois USA; ^3^ Department of Molecular Genetics Leibniz Institute of Plant Genetics and Crop Plant Research (IPK) Gatersleben Seeland Germany; ^4^ Sibelius Natural Products Health Wellness and Fitness Oxford UK; ^5^ Advanced Technologies Cambridge Cambridge UK; ^6^ Quantitative Genetics British American Tobacco Cambridge UK; ^7^ Université de Lorraine AgroParisTech, INRA, Silva Nancy France

**Keywords:** Arabidopsis, drought tolerance, flowering time, plant phenotyping, quantitative trait loci (QTL), water productivity, water use, water‐use efficiency

## Abstract

Natural selection driven by water availability has resulted in considerable variation for traits associated with drought tolerance and leaf‐level water‐use efficiency (*WUE*). In Arabidopsis, little is known about the variation of whole‐plant water use (PWU) and whole‐plant *WUE* (*transpiration efficiency*). To investigate the genetic basis of PWU, we developed a novel proxy trait by combining flowering time and rosette water use to estimate lifetime PWU. We validated its usefulness for large‐scale screening of mapping populations in a subset of ecotypes. This parameter subsequently facilitated the screening of water use and drought tolerance traits in a recombinant inbred line population derived from two Arabidopsis accessions with distinct water‐use strategies, namely, C24 (low PWU) and Col‐0 (high PWU). Subsequent quantitative trait loci mapping and validation through near‐isogenic lines identified two causal quantitative trait loci, which showed that a combination of weak and nonfunctional alleles of the *FRIGIDA (FRI)* and *FLOWERING LOCUS C (FLC)* genes substantially reduced plant water use due to their control of flowering time. Crucially, we observed that reducing flowering time and consequently water use did not penalize reproductive performance, as such water productivity (seed produced per unit of water transpired) improved. Natural polymorphisms of *FRI* and *FLC* have previously been elucidated as key determinants of natural variation in intrinsic WUE (δ^13^C). However, in the genetic backgrounds tested here, drought tolerance traits, stomatal conductance, δ^13^C. and rosette water use were independent of allelic variation at *FRI* and *FLC*, suggesting that flowering is critical in determining lifetime PWU but not always leaf‐level traits.

## Abstract 2

Accelerated flowering time achieved through nonfunctional and weak alleles of flowering time genes *FRIGIDA* (*FRI*) and *FLOWERING LOCUS C* (*FLC*) reduce water use in Arabidopsis thaliana without detrimentally impacting reproductive output.

## 1 INTRODUCTION

Water availability is essential for the optimal allocation of resources to achieve maximal growth and reproductive fitness (Anderson, 2016). Consequently, a water deficit may force survival trade‐off costs resulting in reduced reproductive fitness (Sletvold & Ågren, 2015; Von Euler, Ågren, & Ehrlén, 2014). In natural populations, adaptations to water deficits encompass several unique ecological strategies that include drought escape and avoidance leading to drought resistance. Although drought escape is characterized by rapid growth and early flowering to reproduce before the onset of terminal drought, avoidance limits growth during periods of dehydration through lowering stomatal conductance and transpiration (Ludlow 1989; Kooyers, 2015). Drought resistance traits, characterized by the ability to survive a water deficit, have traditionally been used to assess plant performance under reduced water availability. However, the usefulness of drought resistance as a trait to optimize plant productivity has been questioned, as the improvement of various drought resistance‐related traits has been demonstrated to reduce productivity under some circumstances, regardless of the ability of plants to survive the period of drought stress (Blum, 2005, 2009; Passioura, 2007). It is widely accepted that drought resistance facilitates plant survival, but it does not contribute towards the maintenance of yield following drought stress or in water replete conditions (Blum, 2005, 2009; Passioura, 2007). The identification of plant varieties that are able to produce stabilized or improved yields with reduced water inputs is therefore an important goal for plant breeders, physiologists, and molecular biologists alike (Morison, Baker, Mullineaux, & Davies, 2008; Parry, Flexas, & Medrano, 2005).

Water‐use efficiency (WUE) at the leaf level is the net amount of CO_2_ fixed per unit of transpired water, hereafter referred to as instantaneous water‐use efficiency (*WUE*_i_, *A*/*E*) (Condon, Richards, Rebetzke, & Farquhar, 2004; Table 1). It relates equally to water loss by transpiration and net carbon gain achieved via gas exchange (Long, Marshall‐Colon, & Zhu, 2015). Alternatively, carbon isotope composition (δ^***13***^C; Table 1), as an estimator of intrinsic WUE, that is the ratio of net CO_2_ assimilation to stomatal conductance for water vapour (*A/g*_*s*_; Farquhar & Von Caemmerer, 1982; Farquhar, Ehleringer, & Hubick, 1989), is regularly used to describe integrated leaf‐level intrinsic WUE and have been targeted in several studies as a primary trait to achieve “more crop per drop” as well as enhancing drought resistance (Blum, 2009; Morison et al., 2008).

**Table 1:** Glossary of water use efficiency and water use parameters

Parameter	Abbreviation	Calculations
Carbon isotope composition	δ^13^C	$\frac{13C}{12C}$
Instantaneous leaf‐level water use efficiency	*WUE*i	$\frac{A}{E}$
Absolute vegetative (rosette) water use	VWU	slope 1 of linear regression $\text{slope}=\frac{\text{rSWC}}{\mathrm{day}}-\text{intercept}$
Calculated plant water use	cPWU	VWU * days of flowering
Measured plant water use	mPWU	∑daily added water
Mean daily water use	‐	average of daily added water over the life time of the plant
Water productivity calculated or measured	cWP/mWP	$\frac{\text{seed biomass}}{\text{cPWU}\vee \text{mPWU}}$
Transpiration efficiency calculated or measured	cTE/mTE	$\frac{\text{above ground biomass}}{\text{cPWU}\vee \text{mPWU}}$
Dehydration plasticity (VWU plasticity)	DP	segmented regression $\frac{\left(\text{slope}1-\text{slope}2\right)}{\text{slope}1}$

Abbreviations: A: carbon assimilation; C: carbon; cPWU: calculated lifetime plant water use E: evaporation; mPWU: measured plant water use; rSWC: relative soil water content; VWU: vegetative water use; WUE: water‐use efficiency.

The value of leaf‐level WUE estimates for improving crop yield has previously been questioned. For example, it has been shown that despite the association between δ^***13***^C and WUE in many species (Farquhar et al., 1989), its relation to yield across multiple environments and genotypes is often variable (Condon et al., 2004). This suggests that both additional intrinsic plant factors, as well as environmental conditions, impact the relationship between intrinsic WUE and agronomic WUE, that is, the amount of yield produced per unit of water transpired. Therefore, leaf‐level intrinsic WUE estimates may not be a useful proxy to select for yield under water limited conditions. This lack of consistent upscaling from leaf‐ to whole‐plant WUEs may be a product of the heterogeneity of net CO_2_ assimilation rates within and across individual photosynthetic organs or it may also be due in part to the lack of integration of night‐time transpiration and plant respiration rates in leaf‐level WUE measurements (reviewed in Cernusak, Winter, & Turner, 2009; Cernusak et al., 2013). Furthermore, this inconsistency may be related to changes in environmental conditions leading to variations in other processes that affect CO_2_ supply and demand (Medrano et al., 2015; Seibt, Rajabi, Griffiths, & Berry, 2008). In addition, discrepancies may occur due to genotypic variation in carbon isotope signatures of crop plants being often driven by variation in stomatal conductance (Blum, 2005; Marguerit et al., 2014; Monclus et al., 2006; Monneveux, Sánchez, Beck, & Edmeades, 2006), thereby limiting carbon assimilation and productivity. It should be noted, however, that in some species, variation in δ^13^C has also been attributed to variation in carbon fixation as well as stomatal conductance (Brendel et al., 2008; Donovan, Dudley, Rosenthal, & Ludwig, 2007; Masle, Gilmore, & Farquhar, 2005).

Investigating the natural variation in whole‐plant WUE and the mechanisms of drought resistance in natural populations is challenging, due to difficulties in recreating realistic drought conditions in an experimental setting. For example, in short‐dehydration experiments (Bechtold et al., 2010, 2016; Ferguson, Humphry, Lawson, Brendel, & Bechtold, 2018), water loss is greater in larger plants creating substantial heterogeneity in the timing of water deficits (Kooyers, 2015). Although plant size greatly contributes to water loss in Arabidopsis, drought response traits are independent of the transpiring leaf surface (Ferguson et al., 2018). This suggests that above ground biomass impacts water use and consequently whole‐plant WUE but not necessarily drought tolerance. Central to the determination of whole‐plant WUEs, such as transpiration efficiency (TE, here ratio between aboveground biomass and transpired water; Table 1) or water productivity (WP, here ratio between seed biomass and transpired water; Table 1), is the quantification of water lost by the plant. We have previously shown that leaf‐level *WUE* is not representative of absolute vegetative (rosette) water use (VWU), or biomass production (Ferguson et al., 2018), as the transpiring leaf surface is a major upscaling factor. Additionally, we have demonstrated in a few selected ecotypes that differences in life‐time plant water use (PWU; Table 1) and plant‐level WUE (TE and WP) exist (Bechtold et al., 2010); however, little is known about the underlying molecular mechanisms of the variation in PWU and TE/WP. In Arabidopsis, the measurement of lifetime PWU has received little attention, mainly due to the difficult and time‐consuming nature of manually phenotyping PWU on a daily basis for the majority of the lifetime of the plant (Bechtold et al., 2010, 2013). As plants begin to develop stalks and flowers, automated watering systems (Granier & Tardieu, 2009; Tisné et al., 2013) would cause considerable disturbance of the tall structures. Conversely, nonconveyor belt platforms (Halperin, Gebremedhin, Wallach, & Moshelion, 2017) or a manual approach involving careful handling of flowering plants limits the potential for harmful effects occurring due to movement and touch induced changes (Van Aken et al., 2016). From limited studies of this nature, the C24 ecotype has emerged as drought tolerant and highly water use efficient (Bechtold et al., 2010); additionally, it demonstrates resistance to numerous abiotic and biotic perturbations (Brosché et al., 2010; Lapin, Meyer, Takahashi, Bechtold, & Van den Ackerveken, 2012; Lapin et al., 2012; Xu et al., 2015; Bechtold, Ferguson, & Mullineaux, 2018).

Our recent study of 35 Arabidopsis ecotypes confirmed the above‐described uniqueness of C24 in uniting several desirable water use and drought response traits (Ferguson et al., 2018). To build upon these findings, we set out to ascertain whether PWU of C24 was reduced compared to other ecotypes and whether this had a heritable and genetically discernible basis. We therefore employed a C24 × Col‐0 recombinant inbred line (RIL) population (Törjék et al., 2006) to identify QTLs that underlie the natural variation of these traits. However, due to the difficulties of manually phenotyping PWU, development of a suitable proxy trait was required to phenotype the mapping population in a high‐throughput manner. Arabidopsis represents an ideal system through which to develop and evaluate the usefulness of proxy traits, such as *WUEi*, δ^***13***^C, flowering time, VWU, and biomass parameters for predicting PWU and whole‐plant WUEs. To this end, we assessed the usefulness of this suite of traits for acting as proxies to predict whole‐plant WUEs (TE and WP; see Table 1) in a set of 12 summer annual ecotypes. A highly accurate proxy trait was subsequently identified and employed in a forward genetic screen for whole‐ PWU traits.

## 2 MATERIALS AND METHODS

### 2.1 Plant material and plant growth

**Figure 1:**
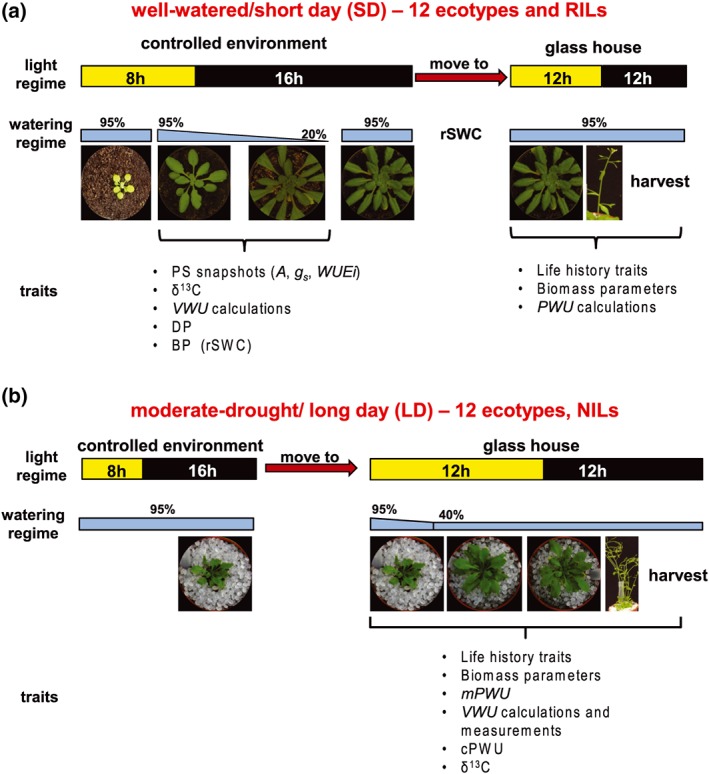
Overview of growth conditions and watering experiments. (**a**) Short‐dehydration experiment carried out on 12 ecotypes and the RIL population. Plants were grown for most of their lifespan under short‐day (65 days) and well‐watered conditions with a short‐dehydration period to assess plant water use and drought sensitivity (b) Continuous maintenance of moderate drought experiment carried out on 12 ecotypes and near‐isogenic lines (NILs). Plants were grown for most of their lifespan under long‐day and moderate drought conditions (40% *rSWC*; Bechtold et al., 2013, 2010). VWU: vegetative water use; PWU: lifetime plant water‐use; DP: dehydration plasticity. See Table 1 for glossary of terms

A selection of 12 facultative summer annual Arabidopsis thaliana (Arabidopsis) ecotypes (Table S1) and 164 RILs derived from a cross between ecotypes Col‐0 and C24 (Törjék et al., 2006) was employed to assess the natural variation of long‐term PWU. The genetic map and genotype information for the RIL population are as described in Törjék et al., 2006 (Table S2). The Col‐0 × C24 RIL mapping population was used to identify QTL relating to key traits associated with water use. Detected QTL regions of interest were further investigated using near‐isogenic lines (NILs) that captured Col‐0 alleles in a homogenous C24 genomic background and vice versa (Törjék et al., 2008). The ecotypes, RILs, and NILs were phenotyped for water use (VWU and PWU), flowering time, and above ground biomass parameters. Additionally, the 12 ecotypes and NILs were phenotyped for δ^***13***^C (Figure 1).

Plants were sown in peat‐based compost (Levington F2 + S, The Scotts Company, Ipswich, UK.) and stratified at 4°C in darkness for 4 days. After stratification plants were grown in a growth chamber at 23°C under short‐day (SD; 8 hr:16 hr; light:dark) conditions, under a photosynthetically active photon flux density of 150 ± 20 μmol · m^−2^ · s^−1^ and at 65% relative humidity (VPD of 1 kPa, Figure 1). Plants were transferred to the glasshouse at distinct stages depending on the applied watering regime (see below and Figure 1). Within the glasshouse, the environmental conditions were variable, as temperature and external light cycles fluctuated during the experimental periods. Supplemental lighting was maintained at a minimum photosynthetically active photon flux density threshold of ~200 *μ*mol · m^−2^ · s^−1^ at plant level for a 12‐hr day (long‐day [LD] conditions). Plants were watered according to the different watering regimes (see Figure 1), and their positions within the two growth environments (SD and LD) were changed daily. In this study, we deliberately opted for transitions between SD and LD conditions (growth chamber to glasshouse) without a vernalization period, which resulted in delayed flowering compared to some studies. This decision was taken as physiological measurements (snapshot measurements for *WUEi*) required a minimal rosette size that would normally not be achieved in vernalized plants.

### 2.2 Watering regimes

#### 2.2.1 Short‐term dehydration experiment for the determination of VWU

All lines undergoing a short‐dehydration experiment were grown in the growth chamber in 6‐cm diameter (0.11 L) pots for the determination of VWU as described in Ferguson et al. (2018). Briefly, at 50‐day postsowing, plants were left to progressively dry to 20% relative soil water content (rSWC), at which point they were rewatered and transferred from the controlled environment room to the glasshouse for flowering time determination and seed production. VWU was calculated as the slope of the linear regression of the rate of drying from 95% to 20% *rSWC* (lasting between 10 and 12 days; Figure 1a and Table 1). Plants were transferred to the glasshouse after rewatering and maintained well‐watered to determine flowering time and the number of rosette leaves at bud initiation. Plant biomass components were separated and measured as rosette biomass (vegetative biomass), chaff biomass (stalks and pods; reproductive biomass), and seed yield (reproductive biomass), and the sum of all biomass components produced the total above ground biomass value. PWU was calculated as VWU multiplied by the time it took from germination to flowering to generate calculated lifetime PWU (cPWU; Table 1). WP was calculated as seed biomass divided by either calculated or measured lifetime water use (cWP or mWP, Table 1). This watering regime is designated as SD, as plants spend most of their life time under SD conditions (~65 days).

#### 2.2.2 Continuous maintenance of moderate drought for determination of lifetime PWU

For the determination of PWU, 8‐cm diameter (0.3 L) pots were filled with the same volume of soil following the experimental setup as described in Bechtold et al. (2010). The soil surface was covered with 0.4‐cm diameter polypropylene granules to limit soil evapotranspiration. Plants were germinated in the previously described growth chamber before being transplanted into individual pots 12 days after sowing at the initiation of the rosette growth stage (Boyes et al., 2001). Four days after being transferred into individual pots, plants were moved into the glasshouse, where pots were weighed daily (Kern PCB, 350‐3 balance) to determine and maintain the pots at a moderate drought level of 40% rSWC (Bechtold et al., 2010). Daily water use was recorded after plants were transferred into the glass house. Control pots without plants were also measured daily to estimate evaporation from the soil surface. Estimates of PWU were corrected to take account of soil evaporation. Flowering time and number of leaves at bud initiation were recorded, and once the final flower had opened, watering ceased, and plants were bagged for harvesting. During harvest the vegetative (rosette) and reproductive (stalks, pods, and seeds) biomass components were separated. Measured PWU (mPWU) was determined as the sum of water added every day until bagging minus the water lost through evaporation from control pots. This parameter is also termed mPWU in order to distinguish it from cPWU (Table 1). This watering regime is denoted as LD, as plants only spend 16 days from germination under SD conditions; the remaining time plants were grown under LD conditions (Figure 1b).

### 2.3 Estimating drought sensitivity

For analysing in more detail the data used for calculating VWU, we applied the Davies test (Davies, 2002) and segmented regression analysis as part of the segmented package in R (Muggeo, 2017) in order to test (a) for a significant difference in slope parameter and (b) for the breakpoint in the regression. This analysis produced the breakpoint in the drying period and the slopes before (stage 1) and after (stage 2) the breakpoint. VWU plasticity was calculated as the slope before the breakpoint (stage1; supposed to represent transpiration under control conditions) − slope after breakpoint (stage2; supposed to represent transpiration under drought conditions)/slope before breakpoint (stage1). Both breakpoint (in terms of rSWC) and VWU plasticity were used to estimate the drought sensitivity (DS) as per Ferguson et al. (2018).

### 2.4 Physiological measurements

#### 2.4.1 Photosynthetic rate (snapshot measurements) in the short‐dehydration experiment

Instantaneous measurements of net CO_2_ assimilation rate (*A*) and stomatal conductance to water vapour (*gs*) and transpiration rate (E) were taken on leaf 7, using an open gas exchange system (PP Systems, Amesbury, MA, USA). Leaves were placed in the cuvette at ambient CO_2_ concentration (C_*a*_) of 400 μmol/mol, leaf temperature was maintained at 22 ± 2°C and vapour pressure deficit was approximately 1 kPa, and irradiance was set to growth conditions (150 μmol · m^−2^ · s^−1^). A reading was recorded after the IRGA conditions had stabilized (approximately 1.5 min), but before the leaf responded to the new environment (Parsons, Weyers, Lawson, & Godber, 1997). *WUE*i was estimated as A/E.

#### 2.4.2 Delta carbon 13 analysis

The carbon isotope composition (δ^13^C) of bulk leaf material was assessed for the 12 ecotypes comprising the SD experiment (well‐watered samples) and the NILs and parental lines from the continuous moderate drought experiment. The harvested leaves had developed during moderate drought stress (40% rSWC). *δ* ^*13*^ *C* was measured as described in Roussel et al. (2009) and Ferguson et al. (2018). *δ* ^*13*^ *C* was calculated as (*R*_*s*_ − *R*_*b*_)/*R*_*b*_ × 1000, where *R*_*s*_ and *R*_*b*_ represent the ^13^C/^12^C ratio in the samples and in the Vienna Pee Dee Belemnite standard, respectively (Craig, 1957).

### 2.5 Statistical analysis

All statistical analyses were performed within the R software environment for statistical computing and graphics (R Core Team, 2015). Experiments using the RIL population were performed across several blocks over a period of 2 years. Each temporally divided block contained the two parental ecotypes and between 20 and 40 RILs. One‐way analysis of variance (ANOVA) comparison of means tests were performed across all lines and all blocks to determine the existence of experimental block effects that could potentially confound further analysis and the QTL mapping. Best linear unbiased predictors (BLUPs) were extracted using the following general linear mixed model: *Y* = *E* + *B* + Residual (Error) variance, where *Y* represents the phenotypic trait parameter of interest and both *E* (Ecotype) and *B* (Experimental block) are treated as random effects, while controlling for fixed effects, that is, "temporal block effects (Lynch & Walsh, 1998). Predicted means were obtained for each trait and for each RIL by adding the appropriate BLUP value to the population mean. Predicted means were employed for all subsequent analyses involving the RILs and for QTL mapping. The general linear mixed models allowed for the determination of phenotypic (*V*_P_) and genotypic (*V*_G_) variation for all trait parameters. These parameters were used to obtain estimates of broad sense heritability (*H* ^2^) as *V*_G_/*V*_P_.

### 2.6 QTL Mapping

We mapped for QTLs underlying all assessed parameters using the qtl R package (Broman & Shen, 2009; Broman, Wu, Sen, & Churchill, 2003). The Lander‐Green algorithm (Lander & Green, 1991), that is, the hidden Markov model technology, was used to reestimate the genetic map using the Kosambi map function to convert genetic distance into recombination fractions with an assumed genotyping error rate of 0.0001. The reestimated genetic map, based on the lines incorporated in this study, was preferred to the original genetic map, which was based on over 400 RILs. The hidden Markov model technology and Kosambi map function were further employed to calculate the probabilities of true underlying genotypes at pseudo‐marker points between actual markers based on observed multipoint marker data, while allowing for the same rate of genotyping errors. Genotypes were calculated at a maximum distance of 2 cM between positions.

Multiple QTL mapping (MQM) was performed using the predicted means derived from BLUPs. The best multiple QTL models were fitted via the multiple imputation approach, using genotype probabilities at both genetic markers and calculated pseudo‐markers. This is the most appropriate method for fitting multiple QTL models, especially when maker density is not especially high (average inter‐marker distance here: 3.87 cM; Broman & Sen, 2009).

About 10 000 permutations were used to determine logarithm of the odd (LOD) significant thresholds for incorporating both additive QTL and epistatic interactions at an experiment‐wise α = 0.05. Automated stepwise model selection was performed (Manichaikul, Moon, Sen, Yandell, & Broman, 2009). The penalties for the stepwise model selection were derived from a two‐dimensional genome scan. Finally, the positions of detected QTLs were refined, and the model was fitted with ANOVA to calculate the effect size, percentage variance explained, and the LOD score for each QTL. Interval estimates of all detected QTLs were obtained as 95% Bayesian credible intervals.

Following MQM, the log_10_ ratio comparing the full QTL model and the single QTL model from the two‐dimensional genome scan was directly assessed to test for the presence of an epistatic interaction between the two main effect QTL for cPWU (Broman & Sen 2009).

To determine whether flowering time, vegetative biomass, or VWU were confounding the results of QTL mapping for cPWU, we performed standard interval mapping to detect QTL for cPWU fitted with multiple imputation and whilst independently including these three traits as covariates in the interval mapping model. This was achieved using the scanone() function within R‐qtl, where the trait covariate, that is, flowering time, vegetative biomass, or VWU, was defined using the “intcovar” argument. About 10 000 permutations were performed to determine the LOD threshold for significance at the 5% level (Broman & Sen 2009). If either of the covariate traits reduced the LOD score, or eliminated the significance, of any of the cPWU QTL, this was interpreted as a confounding effect of that covariate trait on cPWU, such that that QTL could not be described as acting on cPWU in a manner independent of the covariate trait.

### 2.7 Genotyping using insertion‐deletion markers

Insertion‐deletion (InDel) marker polymorphic between Col‐0 and C24 alleles of *FRI* and *FLC* were obtained to address the hypothesis that these genes underlie the two major QTLs detected. A 16‐bp deletion in the Col‐0 allele of *FRI* was scored using primers developed by Johanson et al. (2000). A 30‐bp deletion in the Col‐0 allele of FLC was scored using primers developed by Gazzani, Gendall, Lister, and Dean (2003). InDel markers with a single polymerase chain reaction (PCR) band for both InDels (Figure S1a and Table S3) were assayed by quantitative PCR (qPCR) and high‐resolution melting genotyping using the CFX96 Touch Real‐Time PCR Detection System (BIO‐RAD). This information for 138 individuals of the RIL population and both parents was subsequently integrated into the reestimated genetic map (Figure S1b and Table S4).

### 2.8 Analysis of publicly available RNAseq and microarray datasets

Publicly available RNAseq (Xu et al., 2015; GSE61542) and microarray datasets of C24 and Col‐0 (Bechtold et al., 2010, E‐MEXP‐2732) were analysed for differentially expressed genes. These datasets were compared with the protein coding genes within mapping intervals using VENNY (Oliveros, 2007).

### 2.9 RNA extraction and gene expression analysis by qPCR

Leaves of a minimum of four biological replicates were harvested from the NILs and both parental lines at 26= and 43‐day postgermination and frozen in liquid nitrogen. Total RNA was extracted using Tri‐reagent (SIGMA, Aldrich, UK) according to the manufacturer's instructions. For cDNA synthesis, 1 μg of total RNA was treated with RNase‐free DNase (Ambion) according to manufacturer's instructions and reverse transcribed as previously described (Bechtold et al., 2008). Quantitative real‐time PCR was performed using a cybergreen fluorescence based assay as described previously (Bechtold et al., 2008). Gene‐specific cDNA amounts were calculated from threshold cycle (Ct) values and expressed relative to controls and normalized with respect to Actin and Cyclophilin cDNA according to Gruber, Falkner, Dorner, and Hämmerle (2001). To calculate the standard error of the calculated ratios of fold differences for gene expression data, the errors of individual means were combined “in quadrature,” and the final ratio was a combination of the error of the two‐different means of the NILs and Col‐0 samples. The primers used for RT‐qPCR can be found in Table S3.

## 3 RESULTS

We used a selection of 12 facultative summer annual ecotypes of Arabidopsis that previously demonstrated variation for DS and water use associated traits (Table S1; Ferguson et al., 2018), as well as a RIL mapping population and associated NILs (BC4F3‐4) to examine natural variation of PWU and above ground biomass allocation (Tables S2 and 5). The assessment of natural variation for VWU, PWU, biomass accumulation, and DS was followed by QTL mapping to establish the genetic basis of these traits. Two experimental setups were used as part of this study: (a) 12 ecotypes and RILs—a short‐dehydration experiment under predominantly SD conditions to measure a range of leaf‐level *WUE* parameters (*WUE*i, δ^13^C), VWU, flowering time, biomass parameters, and DS (Figure 1a; Ferguson et al., 2018) and (b) 12 ecotypes and NILs—a continuous moderate drought experiment under predominantly LD conditions, during which *rSWC* was maintained at moderate drought levels (~40% *rSWC*) to measure leaf‐level *WUE* parameters (δ^13^C), VWU, PWU, flowering time, and biomass parameters (Bechtold et al., 2010; Figure 1b).

### 3.1 Identification of a proxy trait for lifetime (plant) water use (PWU)

We analysed a range of parameters associated with plant water status by performing a short dehydration as well as a continuous maintenance of moderate drought experiment on 12 selected Arabidopsis ecotypes (Figure 1 and Table 1). We determined VWU (Ferguson et al., 2018; Figure 1a and Table 1), lifetime PWU (Figure 1b and Table 1), flowering time, above ground biomass parameters, δ^13^C, and calculated whole‐plant WUE parameters, namely, TE and WP (Table 1 and Figure 1; Bechtold et al., 2013, 2010, 2016; Ferguson et al. 2018). Both δ^13^C *and WUEi* measurements were taken to determine the influence of leaf‐level processes on whole plant traits (i.e., transpiring leaf surface area); however, we did not observe a significant relationship with whole‐plant WUE parameters such as TE and WP (Figure S2). We continued to focus on the determination of lifetime PWU and the genetic dissection of PWU and productivity traits, instead of the leaf‐level *WUE* parameters, δ^13^C and *WUEi*.

**Figure 2:**
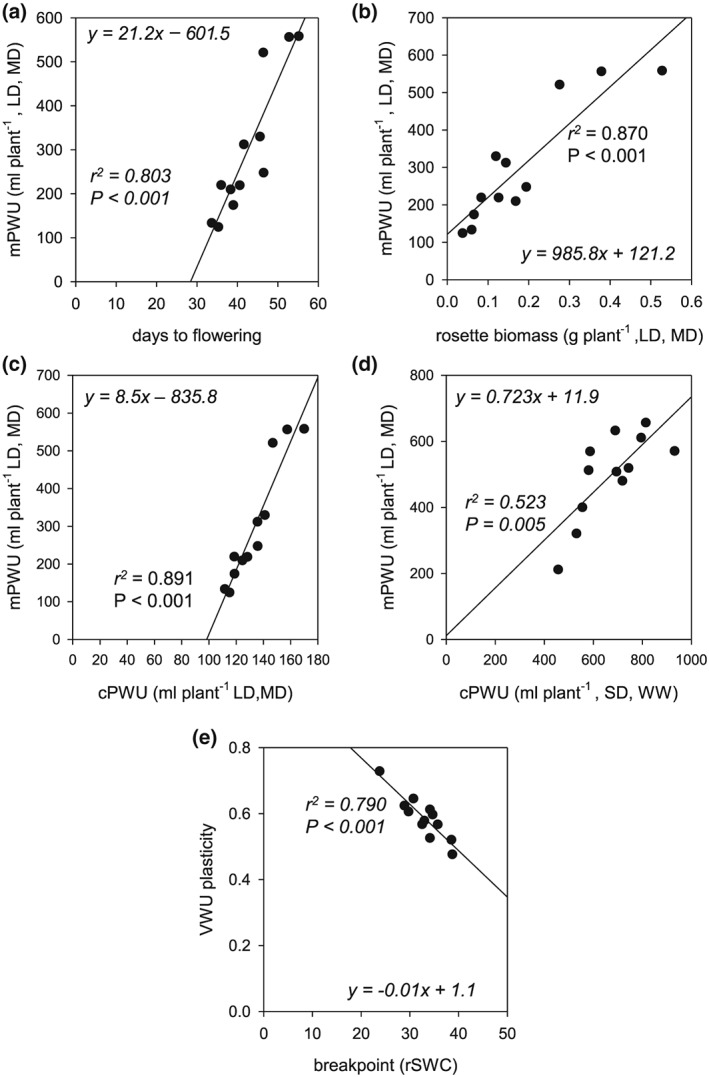
Lifetime water‐consumption and performance parameters in 12 selected ecotypes. (a) Relationship between days to flowering and measured plant water use (mPWU), (b) relationship between vegetative biomass and mPWU, (c) relationship between calculated lifetime plant water use (cPWU) and mPWU within the same experiment, and (d) relationship between cPWU and mPWU between two independent experiments: long‐day, moderate‐drought (LD, MD), and short‐day, well‐watered (SD, WW). The lines represent the equation of the linear regression model, and (e) relationship between the breakpoint in dehydration response and vegetative water use (VWU) plasticity. The *P*‐value of the slope parameter and adjusted *r* ^*2*^ value associated with the linear model are provided for each association

Our usual approach of a manual determination of PWU (Figure 1b) requires the weighing and watering of individual pots until the terminal flower has opened (Bechtold et al., 2010). The manual determination of PWU is challenging and time‐consuming (see Section 1); thus, to facilitate large‐scale manual screening of PWU of the mapping population, we first set out to identify an adequate proxy. We compared biomass production, flowering time, VWU, and PWU between the short‐dehydration and continuous moderate drought experiment carried out on the 12 Arabidopsis ecotypes (Figure 1). The continuous moderate drought experiment revealed that measured PWU (mPWU) was significantly correlated with both flowering (Figure 2a) and vegetative (rosette) biomass (Figure 2b and Tables S6 and S7). Based on these relationships, we developed the proxy parameter “calculated life time (plant) water‐use (cPWU),” as a product of VWU and flowering time:

$$\mathrm{VWU}\;x\;\text{days to flowering}=\text{cPWU}\;\left(\mathrm{see}\;\text{Table}\;1\right)$$

The continuous moderate drought experiment allowed us to directly relate mPWU with cPWU, which showed a highly significant positive correlation within the experiment (Figure 2c). In addition, the correlation between mPWU with cPWU was tighter than the correlations with rosette biomass and flowering time (Figures 2a,b). Importantly, a significant correlation between calculated and measured PWU was also observed when comparing mPWU from the continuous moderate drought experiment under LD conditions, with cPWU of a short‐dehydration experiment under SD conditions (Figure 2d). Therefore, we reasoned that PWU calculated from flowering time and VWU in a short‐dehydration experiment would provide a robust estimate of mPWU.

Furthermore, the short‐dehydration approach allowed us to quantify the drought responses of individual ecotypes by calculating the threshold at which plants enter drought stress (breakpoint) and the plasticity of the drought response (VWU plasticity; Ferguson et al., 2018). The breakpoint negatively correlated with the VWU plasticity, indicating that lines responding to drought stress at higher rSWC showed less absolute change in transpiration throughout the dehydration period and therefore exhibited reduced VWU plasticity (Figure 2e). Therefore, a short‐dehydration experiment allowed us to not only screen and dissect the genetic basis for the natural variation of cPWU and biomass but also assess drought response parameters at the same time.

### 3.2 The genetic dissection of cPWU, drought response, and biomass parameters

Short‐dehydration experiments (Figure 1a) were subsequently performed on 163 individuals of the Col‐0 × C24 RIL population (Table S2) including both parents. To control for experimental block effects, BLUPs were extracted and predicted means were calculated for all traits. The variation in predicted means for all traits was not significantly different from what would be expected of a normal distribution (*P* > 0.05; Kolmogorov–Smirnov normality test), and all traits demonstrated transgressive segregation (Figure S3). We calculated genetic variance (*V*_G_), total phenotypic variance (*V*_P_), and broad sense heritability (*H* ^2^), where all 13 traits assessed demonstrated variation that had a significant heritable basis within the RIL population (Table 2).

**Table 2:** Genotypic and phenotypic variation of the 12 traits assessed as part of the QTL mapping

Trait	*Mean*	*SE*	*V*_*G*_	*V*_*P*_	*H* ^*2*^	*Sig*.
VWU	8.6	0.02	0.49	0.84	0.58	***
Flowering time	74.3	0.4	132.2	170.1	0.78	***
VWU plasticity	0.55	0.03	<0.00	0.01	0.17	***
Breakpoint (day)	5.9	0.16	0.64	2.14	0.30	***
Breakpoint (rSWC)	39.84	0.33	38.41	136.07	0.28	***
Rosette biomass	0.32	0.01	0.02	0.04	0.63	***
Slope 1	−11.28	0.30	0.56	2.59	0.22	***
Slope 2	−5.16	0.27	1.09	2.47	0.44	***
Chaff biomass	0.51	0.01	0.02	0.06	0.36	***
Seed biomass	0.07	0.00	0.00	0.01	0.21	***
Total biomass	0.88	0.0	0.03	0.11	0.29	***
Harvest index	0.04	0.007	0.00	0.00	0.26	***
cPWU	637.8	3.65	9454.7	13404.3	0.71	***

The true (arithmetic) mean, standard error (SE), genetic variance (VG), phenotypic variance (VP), broad sense heritability (*H* ^*2*^), and significance of *H* ^*2*^ (Sig.) are provided for all traits. cPWU: calculated lifetime plant water use; n.s: not significant; rSWC: relative soil water content; VWU: vegetative water use.

*** Significant heritability at the *P* < 0.001 level.

**Figure 3:**
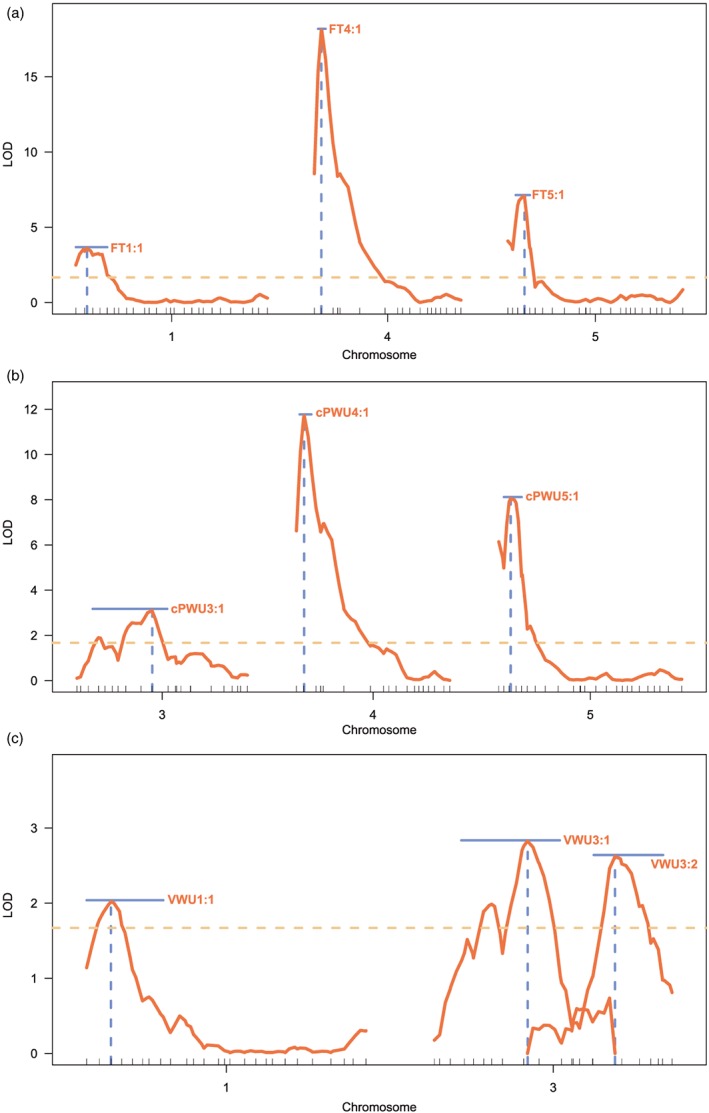
Quantitative trait loci (QTL) mapping. Logarithm of the odd (LOD) profiles for whole chromosomes were significant QTL are located according to multiple QTL mapping. (a) LOD profiles for three significant QTLs underlying variation for flowering time, (b) LOD profiles for three significant QTLs underlying variation for calculated lifetime plant water‐use (cPWU), and (c) LOD profiles for three significant QTL underlying variation for vegetative water use (VWU). The light brown dashed horizontal line indicates the 10% significance threshold for QTL identification. The solid horizontal blue lines indicate the 95% Bayesian confidence intervals of the QTLs. The dashed vertical blue lines indicate the QTL positions on the genetic map [Colour figure can be viewed at wileyonlinelibrary.com]

Adjusted linkage maps were constructed based on the individuals used for mapping. Analyses indicated that 97.5% of the markers had been genotyped for all the RILs, and we observed a virtually even split in the allelic form of these markers, with 50.3% coming from the Col‐0 parental line and 49.7% from the C24 parental line. To identify the genetic variation that causes the observed phenotypic variation in VWU, cPWU*,* flowering time, productivity, and DS traits, MQM was performed (see Section 2) on a minimum of 163 selected individuals. No significant QTL models were identified for seed biomass (Figure S4a), dehydration response (VWU plasticity; Figure S4b), and the breakpoint (Figure S4c). For VWU, FT, cPWU, and slope 1, a total of 10 main effect QTLs were detected (Figures 3 and S4d and Table 3). The percentage of phenotypic variance explained for the cPWU QTLs ranged from 5.24% to 23.16%, for flowering time from 3.64% to 18.09%, and for VWU from 4.25% and 7.32% (Table 3). Because cPWU is calculated based partially on flowering time, there was colocalization between the two main effect cPWU (cPWU4:1 and cPWU5:1) and flowering time QTL (FT4:1 and FT5:1) on chromosomes 4 and 5 (Figure 3a,b, and Table 3). The strong positive correlation observed between flowering time and cPWU suggests that the colocalizing QTLs for these traits were likely to represent the same genes or linkage between causal genes. In general, this suggests that these two major effect cPWU QTLs are fundamentally flowering time QTLs whose effect on cPWU is not independent of flowering time. On the other hand, QTLs detected for VWU did not colocalize with flowering time QTLs (Table 3 and Figure 3). The additional QTL for cPWU (cPWU3:1) located on chromosome 3 is likely a result of allelic variation at the same genes that underlie the VWU3:1 QTL, because cPWU is also calculated based on VWU (Table 3).

**Table 3:** Locations and effect sizes for the significant QTL arising from the QTL mapping via a MQM for water use, harvest index, and flowering time

QTL	Position (cM)	LOD score	Proportion of total genetic variation	95% Bayesian credible interval (cM)	P‐value	Additive genetic effect (SE)
VWU1:1	9.00	1.88	4.25	0.00–28.00	<0.001	0.14 (0.05)
VWU3:1	34.00	3.42	7.90	10.00–46.00	<0.000	−0.19 (0.05)
VWU3:2	68.40	2.51	5.72	58.00–83.37	<0.000	−0.17 (0.05
FT1:1	6.00	3.64	5.51	0.00–16.00	<0.000	−2.74 (0.68)
FT4:1	3.70	18.09	34.93	2.00–6.00	<0.000	6.75 (0.64)
FT5:1	8.00	7.11	11.39	4.00–11.60	<0.000	−3.84 (0.65)
cPWU3:1	38.24	3.04	5.24	8.00–44.00	<0.000	−20.99 (5.77)
cPWU4:1	11.62	11.45	23.16	2.00–8.00	<0.000	47.05 (5.79)
cPWU5:1	7.93	7.04	14.85	2.55–11.60	<0.000	−34.80 (5.76)
Slope3:1	32.61	2.20	6.07	2.00–68.00	<0.001	0.20 (0.06)

The quantitative trait loci (QTL) names are given as the trait followed by the chromosome location. The position in cM, logarithm of the odd (LOD) score (LOD), proportion of total genetic variation, 95% Bayesian credible interval, *P*‐value, and additive genetic effect provided for all significant QTLs.

The three cPWU QTL did not act independently of the trait parameters, from which cPWU is calculated, as confirmed through QTL‐mapping with traits covariates (Figure S5). When performing single QTL‐mapping for cPWU while incorporating flowering time as a covariate in the analyses, the main effect QTL on chromosomes 4 and 5 are not detected; however, the QTL on chromosome 3 that is also detected when mapping for VWU becomes more significant (Figure S5c). Similarly, when incorporating vegetative biomass as a covariate, the effect of these QTL is reduced; however, they are still significant (Figure S5b). Incorporating VWU as covariate removes the importance of the QTL on chromosomes 3 and heightens the significance of the QTLs on chromosomes 4 and 5 (Figure S5d).

The two significant cPWU and flowering time QTLs on chromosomes 4 and 5 (Figure 3a,b) contained two well‐characterized flowering time genes, *FRIGIDA* (*FRI*, chromosome 4; AT4G00650) and *FLOWERING LOCUS C* (*FLC*, chromosome 5; AT5G10140). The ecotype Col‐0 possesses a nonfunctional allele of *FRI* (*fri*) and a functional allele of *FLC* (*FLC*), and the ecotype C24 contains a functional allele of *FRI* (*FRI*) and a weak allele of *FLC* (*flc*; (Johanson et al., 2000; Michaels, He, Scortecci, & Amasino, 2003). A significant epistatic interaction was detected between these QTLs when comparing the full model that incorporates both cPWU4:1 and cPWU5:1 to a single QTL model that only incorporates cPWU4:1 or cPWU5:1 (Figure S6). Transcriptional levels of *FLC* are positively regulated by *FRI* (Deng et al., 2011); thus, the epistatic interaction between these QTL further suggests that *FRI* and *FLC* are the causal genes. InDel markers were designed for both candidate genes and the RIL population was scored for the allelic variant of both genes (see Section 2). This information was incorporated into the genotypic data, and the genetic map was reestimated, which demonstrated that *FRI* and *FLC* were present between the markers that flanked the main effect QTLs on chromosomes 4 and 5, respectively (Figure S1b). The RIL population was subdivided according to the different allelic combination of *FRI* and *FLC* of each individual line (Table S4) to confirm the importance of the functionality of these genes on the traits of interest here.

### 3.3 The genetic action of nonfunctional and weak alleles of *FRI* and *FLC* reduces water use

**Figure 4:**
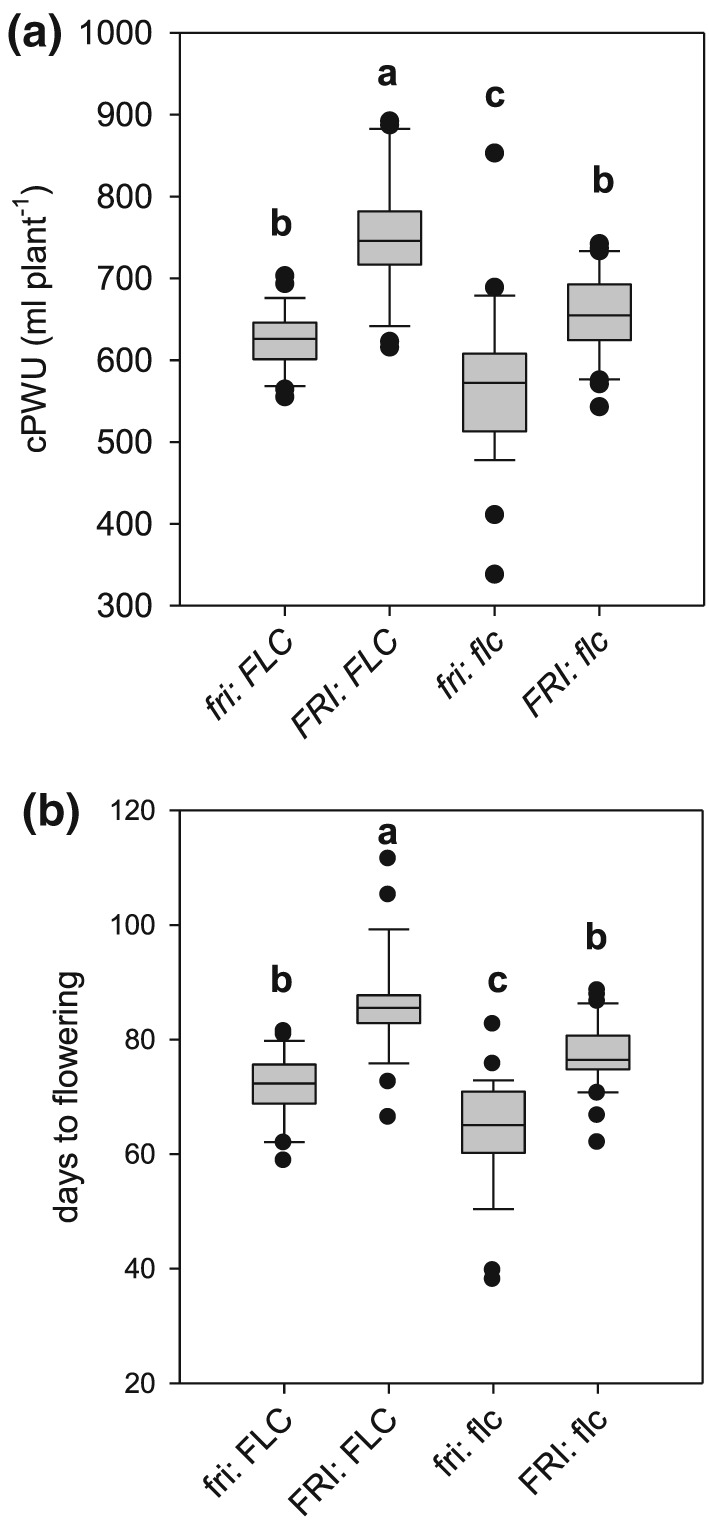
Trait performances of genotypes harbouring different allelic combinations of the FRIGIDA and FLOWERING LOCUS C genes in recombinant inbred lines. Boxplots describing the variation for traits assessed for the four groups based on allelic combination of both *FRI* and *FLC*: (a) cPWU and (b) days to flowering. The letters (a, b, and c) above the boxplot denote the post‐hoc Tukey groups, where allelic groups whose letters are different are significantly different from one another for that trait at *P* < 0.05. The bold line in the centre of the boxplots represents the median, the box edges represent the 25th (lower) and 75th (upper) percentiles, and the whiskers extend to the most extreme data points that are no more than 1.5x the length of the upper or lower segment. Outliers are data points that lie outside the 1.5x interquartile range both above the upper quartile and below the lower quartile

We determined the allelic state of *FRI* and *FLC* in all RILs and divided the population into four groups: (a) *fri*: *FLC* (Col‐0), (b) *FRI*: *FLC,* (c) *fri*: *flc*, and (d) *FRI*: *flc* (C24). One‐way ANOVA comparisons of means and post‐hoc Tukey tests were performed to determine the effect of different allelic combinations on water use and plant development (Figure 4). There were significant and parallel differences in cPWU (Figure 4a) and flowering time (Figure 4b) between the four groups. Possessing nonfunctional and weak alleles of *FRI* and *FLC,* respectively, significantly reduced flowering time and cPWU (Figure 4).

To further test the hypothesis that cPWU is a suitable proxy of mPWU and to confirm that increased life‐span through a combination of *FRI* and *FLC* is the main factor underlying PWU, we subsequently obtained NILs that harboured the Col‐0 allele of *FRI* and *FLC* separately in a homogenous C24 genomic background and vice versa (Table S5). Seven NILs and two parental lines were subjected to a continuous moderate drought experiment, where flowering time, mPWU*,* VWU*,* cPWU, productivity parameters, mean daily water use and δ^13^C and stomatal conductance were determined (Figure 1b). The hypotheses regarding cPWU that emerged from the RIL population were essentially confirmed. The combination of both nonfunctional and weak alleles of *fri* (Col‐0) and *flc* (C24) led to significantly reduced mPWU (Figure S7a) and flowering time (Figure S7b). Due to the significant relationship between flowering time and mPWU (Figure 2a), we assessed whether the different allelic combinations of *FRI* and *FLC* had pleiotropic effects on VWU. There was no significant difference in VWU in both the NILs and RILs under either SD (RILs) or LD (NILs) conditions (Figure S7c,d).

**Figure 5:**
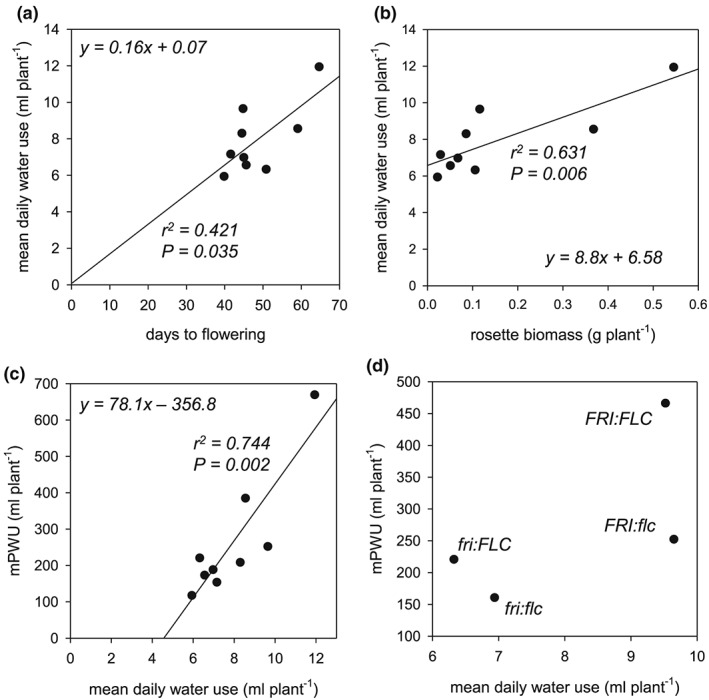
The contribution of mean daily water use in the near‐isogenic lines. (a) Relationship between flowering time and mean daily water use, (b) relationship between rosette biomass and mean daily water use, (c) relationship between mean daily water use and measured plant water use (mPWU), and (d) relationship between mean daily water use and mPWU divided into four *FRI/FLC* allelic groups tested in the near‐isogenic lines. The linear model of the relationship between mean long‐term water use and mean daily water use is provided. *R* ^2^ and *P* values are provided where a significant relationship was identified

Interestingly, we observed a significant relationship between mean daily water use, days to flowering, and rosette biomass in the moderate drought experiments for the 12 ecotypes and the NILs (Figures S8a,b, and 5a,b), leading to high mPWU (Figures S8c and 5c). Therefore, late flowering ecotypes and NILs appear to sustain increased daily water use over a longer period, which was independent of the allelic combinations of *FRI* and *FLC* (Figure 5d).

δ^13^C, while significantly different between Col‐0 and C24, did not show a significant difference among the remaining allelic combinations of *FRI* and *FLC* (Figure S9a), which suggests that δ^13^C was independent of *FRI* and *FLC*. A significant negative correlation between δ^13^C and stomatal conductance indicated that low *g*_*s*_ leads to increased instantaneous *WUE* (A/gs) (*g*_*s*_; Figure S9b; *R* ^2^ = 0.781 *P* < 0.01), which also coincided with the distinct rosette growth phenotype of C24 (Figure S9b,d). In addition, the lack of significant QTLs for VWU, VWU plasticity, and the breakpoint (Figure S4) suggests that leaf‐level drought responses were not genetically controlled in this mapping population and therefore independent of the detected genetic control of flowering time. This was confirmed by the nonsignificant differences in VWU, VWU plasticity, and breakpoint for the four allelic *FRI*/*FLC* groups (Figures S7c and S10a,b).

Importantly, the observation that a combination of *fri* (Col‐0) and *flc* (C24) in the NILs led to significantly reduced mPWU (Figure S7a), and significant variation in δ^13^C (Figure S9a) that did not match the variation for mPWU, supports our observations from the diverse suite of ecotypes. Taken together, this suggests that cPWU is a reliable proxy for mPWU.

### 3.4 Biomass variation and distribution is independent of the genetic action of *FRI* and *FLC*, and growth conditions

**Figure 6:**
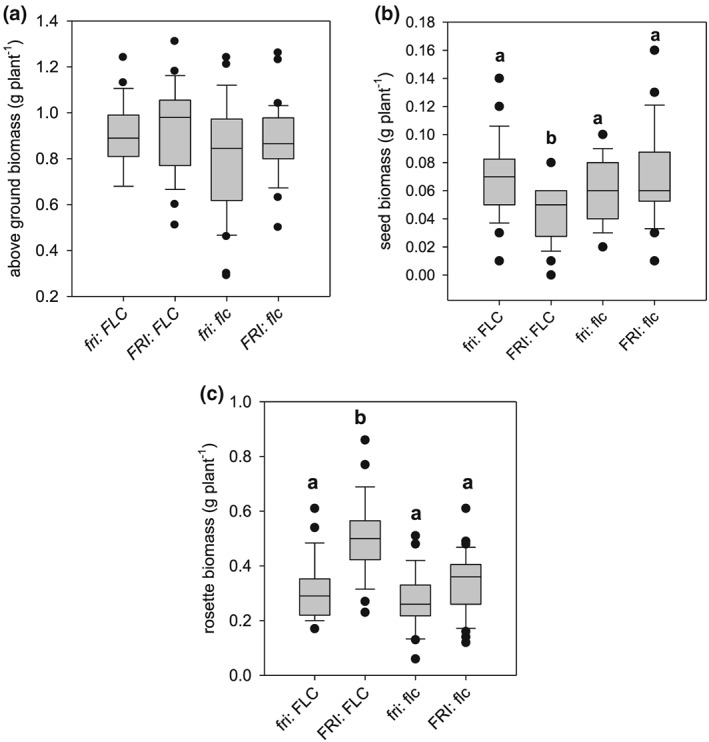
Boxplots of biomass parameters based on allelic combinations of FRI/FLC in the recombinant inbred lines: (a) above ground biomass, (b) seed biomass, and (c) rosette biomass. The letters (a, b, and c) above the boxplot denote the post‐hoc Tukey groups, where allelic groups whose letters are different are significantly different from one another for that trait at *P* < 0.05. The bold line in the centre of the boxplots represents the median, the box edges represent the 25th (lower) and 75th (upper) percentiles, and the whiskers extend to the most extreme data points that are no more than 1.5x the length of the upper or lower segment. Outliers are data points that lie outside the 1.5x interquartile range both above the upper quartile and below the lower quartile

We also assessed whether the different allelic combinations of *FRI* and *FLC* resulting in significantly different PWU had pleiotropic impacts on biomass parameters. For example, the decrease in cPWU in the *fri*: *flc* group did not result in a significant reduction in above ground, seed, or vegetative biomass in the RILs (Figure 6a‐c) or the NILs (Figure S11a‐c), yet the combination of *FRI:FLC* significantly decreased seed and increased vegetative biomass (Figures 6b,c, and S11c). This suggests that the additionally acquired photosynthates acquired by later flowering plants are translocated primarily to vegetative as opposed to reproductive sinks.

**Figure 7:**
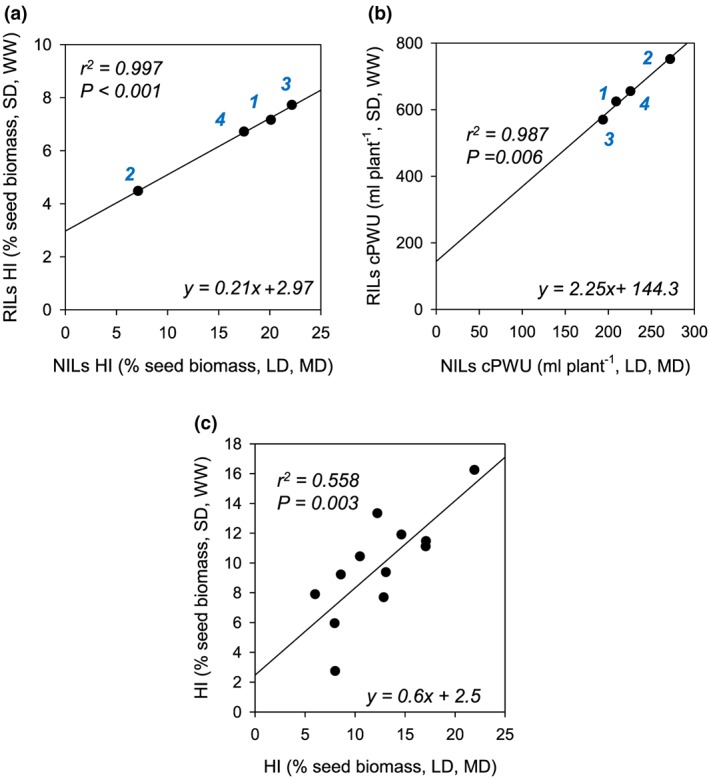
Comparison of water use parameters and harvest index (HI) parameters across different growth and watering regimes. (a) Correlation of HI of the four *FRI/FLC* allelic groups tested in recombinant inbred lines (RILs) and near‐isogenic lines (NILs). RILs were subjected to the growth regime shown in Figure 1A (SD, WW), and NILs were subjected to growth regime shown in Figure 1B (LD, MD). (b) Correlation between cPWU and cPWU of the four *FRI*/*FLC* allelic groups tested in RILs and NILs grown under two different day length and watering regimes (SD, WW and LD, MD). (c) Correlation of HI of 12 ecotypes subjected to the growth different growth regimes shown in Figure 1. The lines represent the equation of the linear regression model. The *P*‐value of the slope parameter and adjusted *R* ^2^ value associated with the linear model are provided for each association. SD: short day; LD: long day; WW: well watered; MD: moderate drought. Allelic combinations: 1: *fri*/*FLC*; 2: *FRI*/*FLC*; 3: *fri*/*flc*; and 4: *FRI*/*flc* [Colour figure can be viewed at wileyonlinelibrary.com]

Biomass allocation (harvest index [HI]) showed substantial variation amongst the NIL and the RIL populations (Figure S12a,b), due to different experimental conditions (SD vs LD, well‐watered vs moderate drought). Despite these experimental differences, relative proportions were highly correlated between the well‐watered and moderate drought experiments (Figure 7), suggesting allelic combinations with low HI in the short‐dehydration experiments (RILs) also showed low HI in the continuous moderate drought experiment (NILs; Figure 7a). Equally, cPWU significantly correlated across the distinct experiments for the different allelic groups (Figure 7b). A similar relationship for PWU and HI across different experiments was also observed in the 12 accessions (Figures 2d and 7c). This suggests that the distribution of biomass and PWU was independent of environmental growth conditions including watering status and day length in both the mapping population and the 12 accessions.

### 3.5 Gene expression

The detected QTL regions contained many genes, as such we explored gene expression differences between the two parents within the mapping intervals for all three mapped traits. This was achieved using a publicly available microarray experiment comparing C24 and Col‐0 (Bechtold et al., 2010) and RNAseq data of both parental accessions (Xu et al., 2015). In total, 9906 protein coding genes were identified within the 95% Bayesian credible intervals (extended to nearest physical markers) on chromosomes 4 and 5 (Table 3), of which 304 showed differential expressions between Col‐0 and C24 (Tables S8 and S9). We randomly selected three to four differentially expressed genes (up and down) for each interval, while also including *FRI*, *FLC*, and *FLOWERING LOCUS T* (*FT*; chromosome 1) for analysis of gene expression in the NILs and both parental lines (Table S10) at 26‐ and 43‐day postgermination.

**Figure 8:**
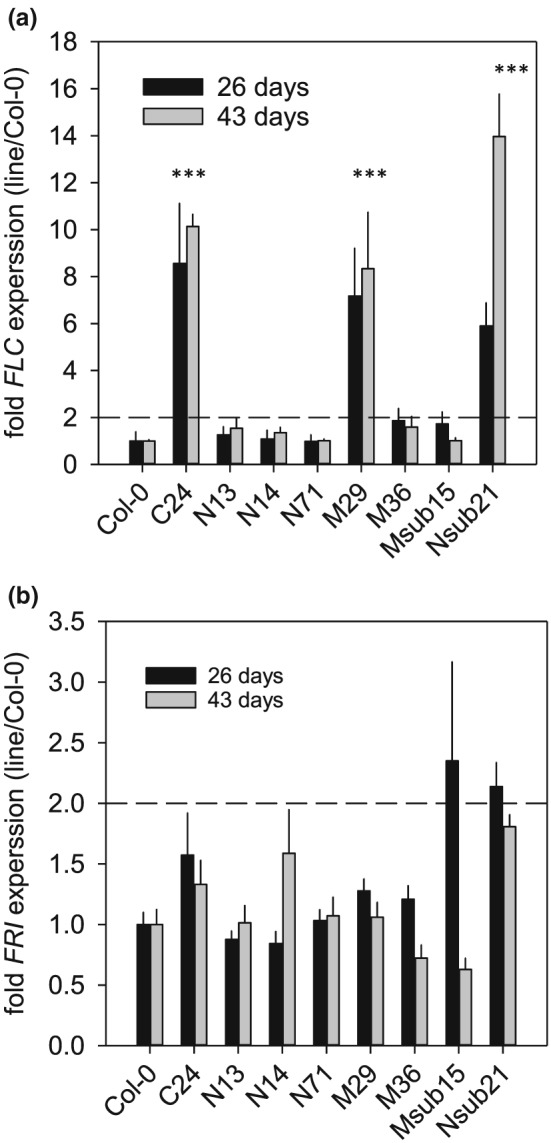
Expression of candidate genes in mapping interval. (a) Gene expression of *FLC* at 26 days after sowing (26 days) and 43 days after sowing (43 days). The stars above the columns denote significant different (*P* < 0.01) expression level compared to Col‐0 at both time points. (b) Gene expression of *FRI* at 26 days after sowing (26 days) and 43 days after sowing (43 days). No significant gene expression levels compared between either the NILs or C24 and Col‐0 were detected

Early studies have shown that *FRI* up‐regulates *FLC* expression in ecotypes that have the active allele of *FRI* (Michaels & Amasino, 1999; Sheldon et al., 1999). NILs carrying the C24 *FRI* allele (Table S5) showed elevated *FLC* expression at 26‐ and 43‐day postgermination in plants grown under SD controlled environment conditions (Figures 1 and 8a). Variation in *FLC* and *FRI* expression at 43‐day postgermination showed a significant association with flowering time and mPWU (Table S10), which was independent of *FT* expression (Table S11). This is in line with QTL mapping results where a significant association of the allelic state of *FRI* and *FLC* with flowering time and PWU was observed under SD controlled environment conditions (Figures 3a,b; 4; and S9a,b). Other highly differentially expressed genes in the mapping intervals on chromosomes 4 and 5 showed no specific pattern that significantly correlated with the flowering time phenotype or mPWU observed in the NILs across the two developmental stages (Table S11).

## 4 DISCUSSION

The ecotype C24 has an unusually rare combination of traits resulting in increased drought resistance, reduced VWU, and increased WP (Bechtold et al., 2010; Ferguson et al., 2018), as well as resistance to a number of other abiotic and biotic stresses (Brosché et al., 2010; Lapin et al., 2012; Xu et al., 2015; Bechtold et al. 2018).

*WUE*_*i*_ is considered to play a key role in PWU (Steduto, Hsiao, & Fereres, 2007) as it relates equally to water loss by transpiration and net carbon gain, thus impacting on biomass production (Steduto et al., 2007; Long et al., 2015). Because of the relationship between leaf and plant‐level *WUE* parameters, high leaf‐level *WUE* is seen as an important trait for minimizing water loss in many different plants species (Blum, 2009; Sinclair & Rufty, 2012; Vadez, Kholova, Medina, Kakkera, & Anderberg, 2014). In addition, *WUE* is often referred to as a drought adaptation trait (Comstock et al., 2005; Condon et al., 2004; McKay et al., 2008) because of the *A*/*g*_*s*_ correlation, where *WUE* can increase during drought stress when stomata close, especially when *A* is not yet proportionally affected (Easlon et al., 2014; Gilbert, Holbrook, Zwieniecki, Sadok, & Sinclair, 2011; Meinzer, Goldstein, & Jaimes, 1984). However, *WUE* only evaluates how much water a plant needs to fix carbon, and in Arabidopsis, where within species variation in *WUE* is predominantly driven by variation in stomatal conductance (Easlon et al., 2014; Ferguson et al., 2018; Vialet‐Chabrand et al. 2016); overall, PWU will therefore be the main driver of *TE*.

### 4.1 The importance of flowering time for plant water‐use strategies

In natural populations, such as Arabidopsis, few studies have compared leaf‐level measurements with whole‐plant estimates of *WUE* (i.e., *TE* or *WP*; Bechtold et al., 2013, 2010; Easlon et al., 2014), and often leaf‐level *WUE* measurements have been exploited as a screening tool to identify genes that could optimize water requirements and yield (Hausmann et al., 2005; Juenger, Mckay, Hausmann, Keurentjes, & Sen, 2005; Masle et al., 2005; McKay et al., 2008; McKay, Richards, & Mitchell‐Olds, 2003). Natural genetic variation for δ^***13***^C has been demonstrated in Arabidopsis (Bouchabke‐Coussa et al., 2008; Easlon et al., 2014; Kenney, Mckay, Richards, & Juenger, 2014; Verslues & Juenger, 2011), and QTL mapping has successfully elucidated the genetic basis of δ^13^C (Ghandilyan et al., 2009; Hausmann et al., 2005; Juenger et al., 2005; Lovell et al., 2015; Masle et al., 2005; McKay et al., 2003; McKay et al., 2008). Interestingly, a positive genetic correlation between flowering time and δ^13^C has been reported (Easlon et al., 2014; McKay et al., 2003), whereas other studies found a negative genetic correlation between flowering time and water content (Loudet, Chaillou, Camilleri, Bouchez, & Daniel‐Vedele, 2002; Loudet, Chaillou, Krapp, & Daniel‐Vedele, 2003). Despite these differences, the link between flowering time and plant water status is undeniable. Furthermore, natural polymorphisms of *FRI* and *FLC* have been identified as key determinants of the natural variation in δ^13^C (Kenney et al., 2014; Lovell et al., 2015; McKay et al., 2003; McKay et al., 2008), and *FLC* is also known to control the circadian rhythm of leaf movement (Edwards et al., 2006). It was therefore suggested that *FLC* may also regulate stomatal transpiration (Edwards et al., 2006), because accessions with a nonfunctional allele of *FLC* showed reduced flowering time and increased water content (Loudet et al., 2002, 2003). Similarly, C24 possess a nonfunctional allele of *FLC* and exhibit a high relative water content and low stomatal conductance (Bechtold et al., 2010; Figure S9a,b). Our data suggest that flowering time achieved through different combinations of weak or nonfunctional alleles of *FRI* and *FLC* explained most of the variation in PWU (Figures 4a and S7a). Leaf‐level traits associated with the lowered stomatal conductance phenotype were independent of variation at these genes (Figure S9a,b). In addition, VWU, average daily water use, or the dehydration response were also not affected by the allelic combinations of *FRI* and *FLC* (Figures 5d; S7c,d; and S10). Accordingly, QTLs identified for VWU did not overlap with the two major intervals containing *FRI* and *FLC* (Figure 3c and Table 2). Importantly, plants with high mPWU also used more water daily, which suggests that lifetime PWU is not only driven by flowering time but also by short‐term water‐use strategies (Figures 5c and S8c).

In this study, cPWU and mPWU was clearly associated with increased flowering time (Figure 2a). Mapping identified three QTLs for cPWU located on chromosomes 3, 4. and 5, and given the observed relationships between lifespan and water use (Figure 2a), two also overlapped with flowering time QTLs (Figure 3 and Table 2). *FRI* and *FLC* were determined to be the causal genes underlying the overlapping QTLs on chromosomes 4 and 5, respectively (Figure S1), which reinforced the role of flowering time in determining lifetime PWU. This is perhaps unsurprising, because a plant that lives for a longer period is likely to use more water; however, this occurred without apparent gain of reproductive biomass (Figures 6b and S11b). Interestingly, other development associated genes such as *ERECTA* (Masle et al., 2005; Villagarcia, Morin, Shpak, & Khodakovskaya, 2012; Shen et al., 2015), *SHORT VEGETATIVE PROTEIN* (*SVP* or *AGL22*; Bechtold et al., 2016), and *HEAT SHOCK TRANSCRIPTION FACTOR A1b* (Bechtold et al., 2013; Albihlal et al., 2018) have been shown to affect stomatal function, stress tolerance, and plant development in Arabidopsis and other plant species.

Similarly, the lack of a significant positive correlation between δ^13^C and flowering time in the NILs suggested that the variation in δ^13^C was independent of *FRI* and *FLC* in this mapping population (Figure S9c). However, increased δ^13^C coincided with reduced stomatal conductance and the distinctive growth phenotype of the C24 rosette (Figure S9b,d). In Arabidopsis, δ^13^C is regulated by variation in stomatal conductance and photosynthetic capacity (Masle et al., 2005), which clearly corroborates the observed link between *g*_*s*_ and δ^**13**^C in the NILs and the independence from *FRI* and *FLC*. C24 is also more drought tolerant compared to Col‐0 based on rosette wilting phenotypes after dehydration (Bechtold et al., 2010), and the drought response parameters were also independent of *FRI* and *FLC* in the RIL population (Figure S10).

### 4.2 The impact of day length on flowering time and water use

Col‐0 is a rapid cycling ecotype (Shindo et al., 2005) and the higher *FLC* expression levels in C24 would suggest a late‐flowering phenotype compared to Col‐0 (Figure 8a). However, early genetic studies have shown that C24 contains an allele of *FLC* that suppresses the late flowering phenotype caused by dominant alleles of *FRI*, whereas Col‐0 contains an allele of *FLC* that does not suppress the late‐flowering caused by dominant *FRI* alleles (Koornneef, Blankestijn‐de, Hanhart, Soppe, & Peeters, 1994; Lee, Michaels, Masshardt, & Amasino, 1994; Sanda & Amasino, 1996). Therefore, we do not see a significant difference in flowering time between Col‐0 and C24 in un‐vernalized plants (Figure 4b). The transition from SD to LD conditions as part of our growing regimes (Figure 1) mimics the natural progression in day length from spring to summer, which is commonly experienced by spring/summer annuals. Despite the difference in day length and watering regimes between the short‐dehydration and moderate drought treatments (Figure 1), PWU and biomass allocation were significantly correlated between experiments (Figure 7). This suggested that even though absolute values for HI and PWU were different the relative difference between lines remained the same (Figure 7), indicating that day length does not alter overall water use and developmental strategies in a genotype‐by‐environment specific manner.

With respect to the above, it is worth noting that subjecting summer or winter annual ecotypes to long photoperiods may result in outcomes that could be problematic especially when assessing mechanisms related to leaf‐level *WUEi* drought resistance strategies, because these are often closely linked to flowering time. For example, Riboni, Galbiati, Tonelli, and Conti (2013) and Riboni, Robustelli, Galbiati, Tonelli, and Conti (2014) demonstrated that the induced drought escape mechanisms in Arabidopsis are promoted by the drought mediated up‐regulation of florigens in an ABA‐ and photoperiod‐dependent manner, so that early flowering (drought escape) can only occur under LDs, independent of *FT* and *CONSTANS*. This is in line with our observation that flowering time and mPWU are associated with *FRI* and *FLC* expression but seemingly independent of *FT* expression (Figure 8 and Tables S10 and S11).

### 4.3 The role of *FRI* and *FLC* in determining water use and biomass allocation

*FRI* and *FLC* respond to seasonal variation in temperature, thus play a crucial role in floral transitioning (Koornneef et al., 1994; Lee et al., 1994; Michaels & Amasino, 2001). *FLC* is a MADS box transcription factor that inhibits the transition to flowering by repressing the expression of floral integrators, such as *FT* and *SUPPRESSOR OF OVEREXPRESSION OF CONSTANS1* (*SOC1*; Hepworth, Valverde, Ravenscroft, Mouradov, & Coupland, 2002; Helliwell, Wood, Robertson, James Peacock, & Dennis, 2006; Deng et al., 2011). Most rapid‐cycling accessions of Arabidopsis contain naturally occurring loss‐of‐function mutations in *FRI* and therefore have low levels of *FLC* expression and are early flowering even in the absence of vernalization (Johanson et al., 2000).

Despite variation in cPWU mapping to *FLC* and *FRI*, we cannot explicitly rule out an indirect effect of flowering time differences on water use (Figure S5c). Especially since *FLC* expression remained high in C24 and two NILs throughout the experiment (Figure 8a), independent of the *FLC* allele present (Table S5). However, the reduction in mPWU attained via introgression of the nonfunctional Col‐0 allele of *FLC* or the functional C24 *FRI* allele into the C24 and Col‐0 genomic background, respectively, demonstrates that although flowering time ultimately impacts PWU, it does not confound the importance of these genes in determining PWU.

Interestingly, two major *FLC* haplogroups were associated with flowering time variation in *Arabidopsis* under field‐like conditions, but only in the presence of functional *FRI* alleles (Caicedo, Stinchcombe, Olsen, Schmitt, & Purugganan, 2004). This is in line with our finding that the functional C24 allele of *FRI* (*FRI*) was required for increased *FLC* expression, even though *FRI* expression was not significantly altered (Figure 8b and Tables S10 and S11). Furthermore, a study of ~150 accessions showed that the role of *FLC* in regulating flowering time is less important under SD conditions (Lempe et al., 2005), which suggests that the impact of *FLC* on PWU in our experiments may have been influenced by the environmental growth conditions such as photoperiod and potentially watering status (Figure 1).

However, because *FLC* also acts in conjunction with other MADS‐box proteins to regulate various aspects of plant development through a large variety of target genes (Deng et al., 2011), and rapid‐cycling accessions contain a number of other genes regulating *FLC* expression, collectively known as the autonomous floral‐promotion pathway (Michaels & Amasino, 1999; Sheldon et al., 1999), we cannot rule out that other genetic factors affecting flowering time may indirectly contribute to the variation in whole PWU. Especially, since, ~50% of the total genetic variation for flowering time was not dissected in this study (Table 3.)

The analysis of such putative relationships was beyond the scope of this study. Yet, the considerable number of *FLC* targets and their involvement in different developmental pathways may reflect an important strategy to integrate environmental signals and plant development to ensure reproductive success under many different conditions.

Short‐term stress‐mediated initiation of flowering pathways also involves the repression of *FLC* expression. Cold or saline stress‐dependent activation of *miR169b* was shown to repress the expression of the *NF‐YA2* transcription factor, which in turn reduces *FLC* expression promoting early flowering (Xu et al., 2014). Here, stress treatments were shown to accelerate flowering (escape response) involving the above‐described signalling cascade. We have previously demonstrated that the experimental watering regimes employed in this study (Figure 1) do not initiate a similar escape response in the progenitors of the mapping population and several other rapid cycling ecotypes (Ferguson et al., 2018; Bechtold et al., 2010; 2013). Heat sensitivity has been associated with late flowering haplotypes in vernalized plants, and *FLC* haplotypes resulting in late flowering showed reduced silique length, suggesting a negative correlation between flowering time and seed productivity (Bac‐Molenaar et al., 2015). This negative correlation corroborates our findings, where late flowering RILs and NILs produced less seed biomass and vice versa independent of photoperiod and watering conditions (Figures 6b and S11b).

However, well‐known work from the previous decade has demonstrated a pleiotropic link between flowering time and δ^13^C (WUE; McKay et al., 2003, Juenger et al., 2005). Similarly, positive phenotypic associations between flowering time and δ^13^C have been reported (Easlon et al., 2014; Kenney et al., 2014). It has therefore been suggested that functional alleles of *FRI* and *FLC* indirectly increase δ^13^C, suggesting that late flowering genotypes have greater WUE (McKay et al., 2003). The other referenced studies here support this notion in terms of flowering time and WUE but not with respect to the allelic state of *FRI* and *FLC*. In this study we have identified *FRI* and *FLC* as underlying major QTLs for flowering time and cPWU. Because cPWU is a factor of flowering time, cPWU4:1 and cPWU5:1 cannot be considered independent of flowering time. Nevertheless, the demonstration of reduced mPWU without compromising reproductive fitness in NILs harbouring nonfunctional and weak alleles of *FRI* and *FLC* (Figure S7a) suggests that accelerating flowering time may be the most efficacious means to improve WUE. However, components of previous work essentially suggest that based on a leaf‐level WUE proxy trait, delaying flowering time will increase WUE (Easlon et al., 2014; Kenney et al., 2014; McKay et al., 2003). Thus, this present study illuminates the importance of assessing water use at the whole plant and life time level.

It is important to note that *FRI* has been identified as playing a major role in determining adaptations to water availability through trait correlations along an axis, where functional *FRI* facilitates dehydration avoidance through elevated WUE (measured as δ13C; Lovell et al., 2013). Conversely, Lovell et al. (2013) demonstrated that reduced expression of *FRI* facilitates a drought escape strategy owing to earlier flowering, which is linked to lower WUE. This finding of Lovell et al. (2013) is partly supported by our results in the sense that *fri* has the capacity to facilitate a drought escape response; however, the short‐dehydration experiment (Figure 1a) does not elicit early flowering in either Col‐0 or C24 (Ferguson et al., 2018). In addition, our results build upon these findings by also highlighting the importance of *FLC*, because possessing *fri* and *flc* reduces water use much more than just possessing one or the other (Figures 4a and S7a). Furthermore, our results demonstrate that this does not come at the cost of reducing reproductive output (Figure 8b), and as a consequence water productivity increases.

### 4.4 The relationship between leaf‐level and whole‐plant measures of water use

Leaf‐level measures of *WUE*, taken during vegetative growth, are not representative of whole plant measures such as *TE* or *WP* (Figure S2a–d). This suggests that plants with improved δ^13^C and/or *WUE*_*i*_ are not necessarily diverting additionally acquired photosynthates toward reproductive growth. In addition, our estimation of *TE* is clearly biased towards the final above ground biomass, neglecting root architecture. It is well established that both root depth and density play a major role in optimizing water uptake depending on the hydrological conditions (Czyz & Dexter, 2012; Falik, Reides, Gersani, & Novoplansky, 2005), but variation here may have been limited due to their likely pot bound nature. However, the relative performance of NILs and ecotypes was highly correlated between different experiments (Figure 7), suggesting that the variation observed for *TE* even though biased may reflect actual genotypic differences.

Different drought resistance mechanisms, such as avoidance by maintaining high plant water status and/or drought escape through early flowering (Levitt, 1985), are critical from an ecological standpoint, facilitating population persistence in regions characterized by frequent and/or extended periods of reduced water availability (Araus, Slafer, Reynolds, & Royo, 2002; Gechev, Dinakar, Benina, Toneva, & Bartels, 2012; Kooyers, 2015; Kooyers, Greenlee, Colicchio, Oh, & Blackman, 2015). However, leaf‐level traits such as high *WUE*_*i*_/δ^13^C aimed at preserving water may not always ensure high productivity, and lifespan also determines water use but not necessarily biomass production (Figures 4, 6, and S11b), or allocation (Figure S12; Ferguson et al., 2018). In late flowering plants, photosynthates are not translocated to reproductive sinks, but instead to vegetative biomass (Figure S2), which either suggests poor resource allocation in late flowering ecotypes or a diversion of resources toward abiotic stress defence mechanisms associated with reduced water availability (Claeys, Inze, & Inzé, 2013). Recent studies on the perennial species Arabidopsis lyrata and 35 Arabidopsis thaliana accessions highlighted that populations increased their reproductive output while reducing vegetative growth (Ferguson et al., 2018; Remington, Leinonen, Leppälä, & Savolainen, 2013), which may be even more prevalent in annual plants that only have one opportunity at reproduction. Although recent reports have clearly shown that there is a selection on early flowering in Arabidopsis due to increased plant fitness (Ågren, Oakley, Lundemo, & Schemske, 2017; Austen, Rowe, Stinchcombe, & Forrest, 2017; Gnan, Marsh, & Kover, 2017), still little is known about the genotype‐to‐phenotype basis of this resource allocation trade‐off.

## 5 CONCLUSION

We conclude that flowering time is an important determinant of lifetime PWU strategies in Arabidopsis, as well as a critical life history trait important for seed production. Additional, absolute water use at the vegetative growth stage contributes to overall PWU, albeit to a much‐reduced degree. The causal genes that underlie VWU QTLs are ambiguous and will require further fine‐mapping. We have demonstrated that Arabidopsis PWU strategies can be independent of traditional leaf‐level measures of drought tolerance, *WUE*, and biomass traits, and consequently, genes identified based on these traditional performance traits may not lead to improved productivity under water limiting or water‐replete conditions.

## Supporting information

Figure S1. The single nucleotide polymorphism (SNP) markers used and their position on the re‐estimated linkage map. **a** ‐ *InDel* markers for *FRI* and *FLC*, used to score the C24 x Col‐0 RIL population, and **b** ‐ Position in cMs of all markers on the re‐estimated genetic map.
Figure S2. Comparison of leaf level water use efficiency and biomass level water use efficiency parameters. **a ‐ b** Relationship between δ^13^C, and whole plant water use efficiency parameters biomass level *WUE* parameters: *TE* (transpiration efficiency) and *WP* (water productivity) and **c – d** Relationship between *WUE*_*i*_, and whole plant water use efficiency parameters biomass level *WUE* parameters, *TE* and *WP*. The associations are not significant in all cases.
Figure S3. Distribution of estimated means for all traits assessed as part of the QTL mapping. **a** ‐ vegetative water use (VWU), **b** ‐ days to flowering, **c** ‐ seed biomass, **d** ‐ calculated lifetime plant water use (cPWU), **e** ‐ dehydration plasticity (VWU plasticity), and **f** ‐breakpoint (rSWC) of the segmented regression. For all traits, a Shaprio‐Wilk test of normality was performed on the estimated means of all RILs, where all traits demonstrated variation that was not significantly different from a normal distribution (*P* > 0.05). Green arrows indicate the position of C24 and red arrows indicate the position of Col‐0. The estimated means for the parental lines are also provided (Red – Col‐0, Green – C24)
Figure S4: Additional QTL mapping results. **a** ‐ LOD profiles for seed biomass, with no significant QTL detected, **b** ‐ LOD profiles for dehydration plasticity, with no significant QTL detected, **c** ‐ LOD profiles for breakpoint (rSWC), with no significant QTL detected, and **d** – LOD profiles for slope 1, with one significant QTL detected. The dashed horizontal red line indicates the 0.05 genome‐wide significance threshold.
Figure S5: Single QTL mapping for calculated plant water use with and without traits as covariates. **a –** Without a trait covariate. **b –** With rosette biomass as a trait covariate. **c –** With flowering time as a trait covariate. **d‐** With vegetative water use as a covariate.
Figure S6: LOD scores for a two‐dimensional genome scan for calculated plant water use. Values in the upper left triangle represent the full QTL model. Values on the lower right triangle represent the likelihood ratio comparing the full model with QTLs on all chromosomes with the single QTL model, thus indicating the presence of epistatic interactions.
Figure S7: Trait performances of genotypes harbouring different allelic combinations of the *FRIGIDA* (*FRI*) and *FLOWERING LOCUS C* (*FLC*) genes. Boxplots describing the variation for traits assessed for the 4 groups based on allelic combination of *FRI* and *FLC,* **a** – mPWU in the NILs, **b** ‐ days to flowering in the NILs, **c** ‐ VWU based on allelic combinations of *FRI/FLC* in the RILs, and **d** ‐ VWU based on allelic combinations of *FRI/FLC* in the NILs. The letters (a, b, and c) above the boxplot denote the post‐hoc Tukey groups, where allelic groups whose letters are different are significantly different from one another for that particular trait at *P* < 0.05. The bold line in the centre of the boxplots represents the median, the box edges represent the 25^th^ (lower) and 75^th^ (upper) percentiles, the whiskers extend to the most extreme data points that are no more than 1.5x the length of the upper or lower segment. Outliers are data points that lie outside the 1.5x interquartile range both above the upper quartile and below the lower quartile.
Figure S8: The contribution of mean daily water use in the 12 ecotypes. **a ‐** relationship between flowering time and mean daily water use, **b ‐** relationship between rosette biomass and mean daily water use, and **c ‐** relationship between mean daily water use and mPWU. The linear model of the relationship between mean long‐term water use and mean daily water use is provided. R2 and P values are provided where a significant relationship was identified.
Figure S9: Phenotype of NILs and parental lines. **a** ‐ boxplots of leaf level *WUE* (δ^13^C) for the 4 groups based on allelic combination of both *FRI* and *FLC* in the NILs and both parents. The letters (a, b) denote the post‐hoc Games‐Howell groups, where allelic groups whose letters are different are significantly different from one another for that trait at *P* < 0.05. The bold line in the centre of the boxplots represents the median, the box edges represent the 25^th^ (lower) and 75^th^ (upper) percentiles, the whiskers extend to the most extreme data points that are no more than 1.5x the length of the upper or lower segment. Outliers are data points that lie outside the 1.5x interquartile range both above the upper quartile and below the lower quartile, **b** ‐ phenotype scoring based on rosette growth (panel C), stomatal conductance (*gs*) and δ^13^C measurements. There was a significant negative correlation between gs and δ^13^C. r^2^ = 0.781, *P* < 0.001, **c** ‐ relationship between δ^13^C and flowering time, and **d** ‐ rosette growth at 25 days post sowing.
Figure S10: Boxplots of drought response parameters derived from segmented regression analysis based on allelic combinations of FRI/FLC. **a** ‐ dehydration plasticity (see Table 1), and **b** ‐ breakpoint (rSWC) between segment 1 and 2. Both parameters were calculated using predicted means of the short dehydration experiment performed on the RIL population. No significant differences were detected between the four allelic combinations. The bold line in the centre of the boxplots represents the median, the box edges represent the 25^th^ (lower) and 75^th^ (upper) percentiles, the whiskers extend to the most extreme data points that are no more than 1.5x the length of the upper or lower segment. Outliers are data points that lie outside the 1.5x interquartile range both above the upper quartile and below the lower quartile.
Figure S11: Boxplots of biomass parameters based on allelic combinations of FRI/FLC in the NILs **a** – above ground biomass, **b** – seed biomass, and **c** – rosette biomass. The letters (a, b, and c) above the boxplot denote the post‐hoc Tukey groups, where allelic groups whose letters are different are significantly different from one another for that trait at *P* < 0.05. The bold line in the centre of the boxplots represents the median, the box edges represent the 25^th^ (lower) and 75^th^ (upper) percentiles, the whiskers extend to the most extreme data points that are no more than 1.5x the length of the upper or lower segment. Outliers are data points that lie outside the 1.5x interquartile range both above the upper quartile and below the lower quartile.
Figure S12: Above ground biomass allocation. **a** ‐ biomass distribution in the NILs of moderate drought stressed plants. **b** ‐ biomass distribution in 164 RILs including both parents.

Table S1: Ecotypes used in benchmarking experiment
Table S2: RIL genotypes according to Tjörék *et al*. (2006)
Table S3: Primers used in genotyping and qPCR
Table S4: Genotyping of *FRI* and *FLC* alleles in RIL population using InDel markers, scored by qPCR and high‐resolution melt (HRM) curve.
Table S5: Genotypes of near isogenic lines (NILs)
Table S6: Correlation matrix of traits analysed for the 12 ecotypes population
Table S7: Correlation matrix of traits analysed for the RIL population
Table S8: Number of differentially expressed protein coding genes in mapping intervals
Table S9: IDs of differentially expressed genes in mapping intervals
Table S10: Fold expression and error (Line/Col‐0) of selected DE genes in three mapping intervals at 26‐ and 43 days post germination (*n* = 3).
Table S11: Association between gene expression and mPWU and flowering time (Flowering). Genes FLOWERING LOCUS T (FT), FRI, FLC and At4g00960.
