# Supplementary material for: Accelerated flowering time reduces lifetime water use without penalizing reproductive performance in Arabidopsis
Source: Plant Cell Environ. 2019 Mar 12;42(6):1847–67. doi: 10.1111/pce.13527 (PMC6563486; doi:10.1111/pce.13527)
Supplement: Supplementary file 1 — Figure S1. The single nucleotide polymorphism (SNP) markers used and their position on the re‐estimated linkage map. a ‐ InDel markers for FRI and FLC, used to score the C24 x Col‐0 RIL population, and b ‐ Position in cMs of all markers on the re‐estimated genetic map. Figure S2. Comparison of leaf level water use efficiency and biomass level water use efficiency parameters. a ‐ b Relationship between δ13C, and whole plant water use efficiency parameters biomass level WUE parameters: TE (transpiration efficiency) and WP (water productivity) and c – d Relationship between WUE i, and whole plant water use efficiency parameters biomass level WUE parameters, TE and WP. The associations are not significant in all cases. Figure S3. Distribution of estimated means for all traits assessed as part of the QTL mapping. a ‐ vegetative water use (VWU), b ‐ days to flowering, c ‐ seed biomass, d ‐ calculated lifetime plant water use (cPWU), e ‐ dehydration plasticity (VWU plasticity), and f ‐breakpoint (rSWC) of the segmented regression. For all traits, a Shaprio‐Wilk test of normality was performed on the estimated means of all RILs, where all traits demonstrated variation that was not significantly different from a normal distribution (P > 0.05). Green arrows indicate the position of C24 and red arrows indicate the position of Col‐0. The estimated means for the parental lines are also provided (Red – Col‐0, Green – C24) Figure S4: Additional QTL mapping results. a ‐ LOD profiles for seed biomass, with no significant QTL detected, b ‐ LOD profiles for dehydration plasticity, with no significant QTL detected, c ‐ LOD profiles for breakpoint (rSWC), with no significant QTL detected, and d – LOD profiles for slope 1, with one significant QTL detected. The dashed horizontal red line indicates the 0.05 genome‐wide significance threshold. Figure S5: Single QTL mapping for calculated plant water use with and without traits as covariates. a – Without a trait covariate. b – With rosette [file PCE-42-1847-s001.pdf]

a

b

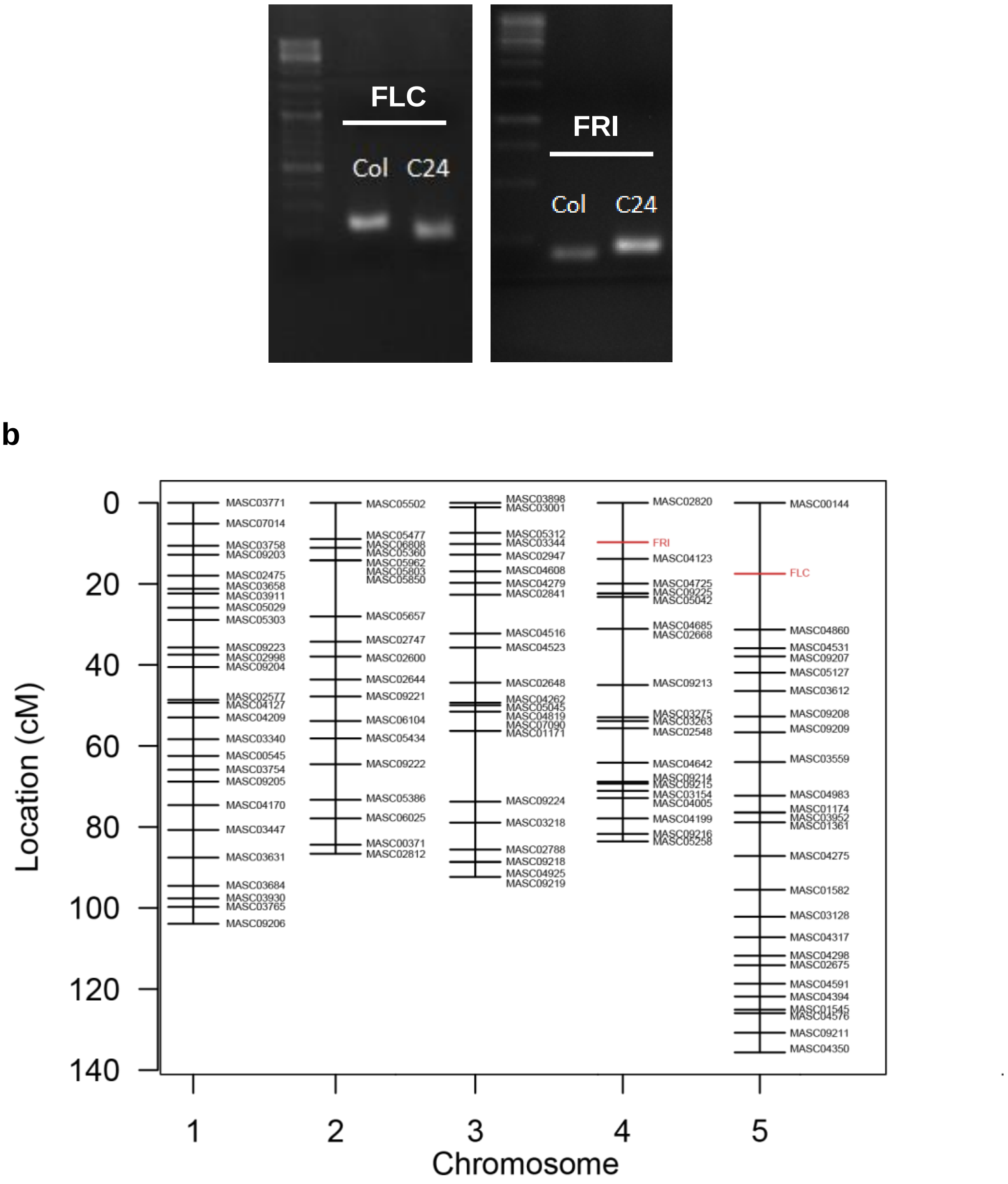

Figure S1. The single nucleotide polymorphism (SNP) markers used and their position on the re-estimated linkage map. a - *InDel* markers for *FRI* and *FLC*, used to score the C24 x Col-0 RIL population, and **b -** Position in cMs of all markers on the re-estimated genetic map.

Supplemental Figure 1

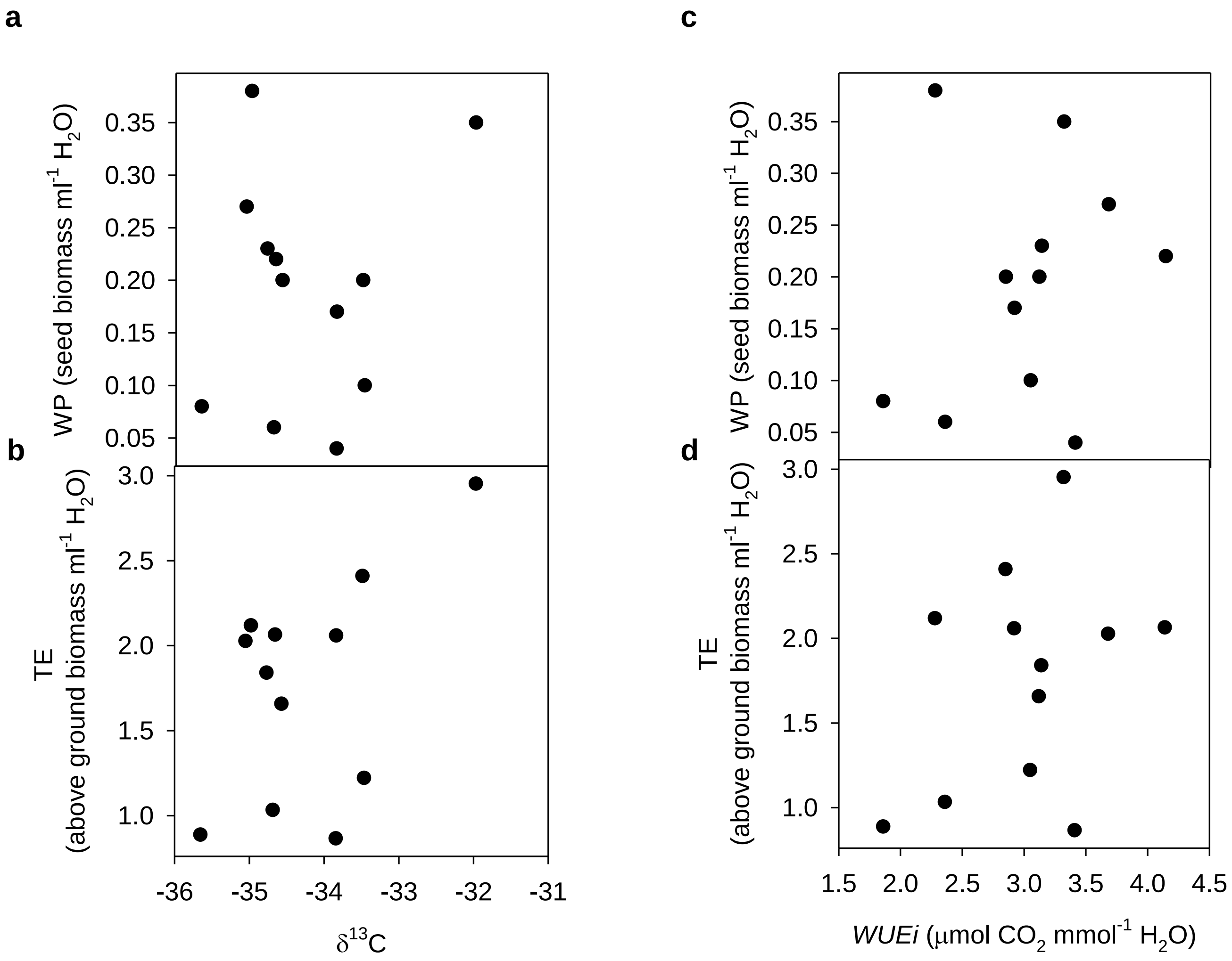

Figure S2. Comparison of leaf level water use efficiency and biomass level water use efficiency parameters. **a - b** Relationship between $\delta^{13}C$, and whole plant water use efficiency parameters biomass level *WUE* parameters: *TE* (transpiration efficiency) and WP (water productivity) and **c – d** Relationship between WUE_i, and whole plant water use efficiency parameters biomass level *WUE* parameters, *TE* and *WP*. The associations and are not significant in all cases.

Supplemental Figure 2

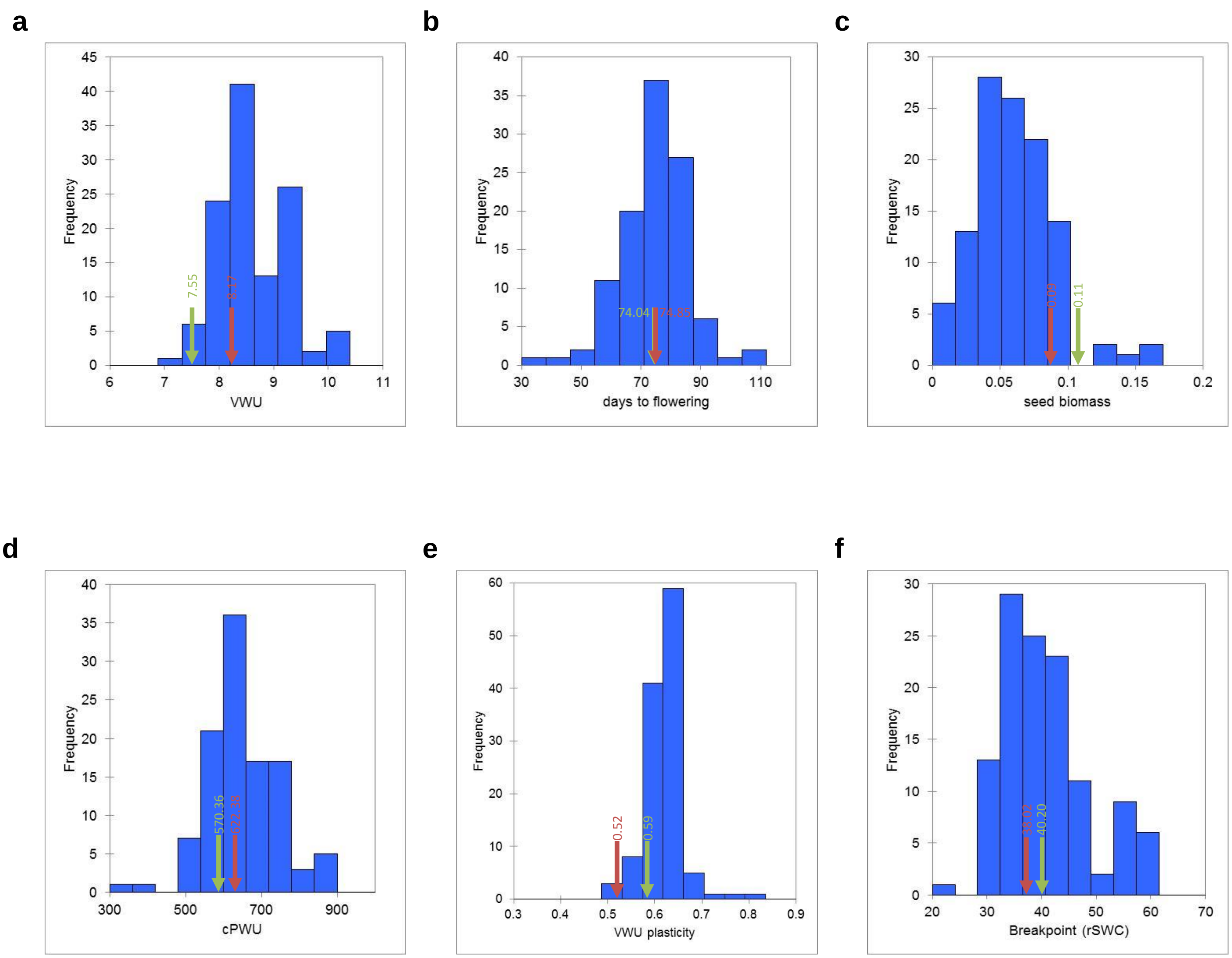

Figure S3. Distribution of estimated means for all traits assessed as part of the QTL mapping. a - vegetative water use (VWU), **b** - days to flowering, **c** - seed biomass, **d** - calculated lifetime plant water-use (cPWU), **e** - dehydration plasticity (VWU plasticity), and **f** -breakpoint (rSWC) of the segmented regression. For all traits, a Shaprio-Wilk test of normality was performed on the estimated means of all RILs, where all traits demonstrated variation that was not significantly different from a normal distribution (P > 0.05). Green arrows indicate the position of C24 and red arrows indicate the position of Col-0. The estimated means for the parental lines are also provided (Red – Col-0, Green – C24)

Supplemental Figure 3

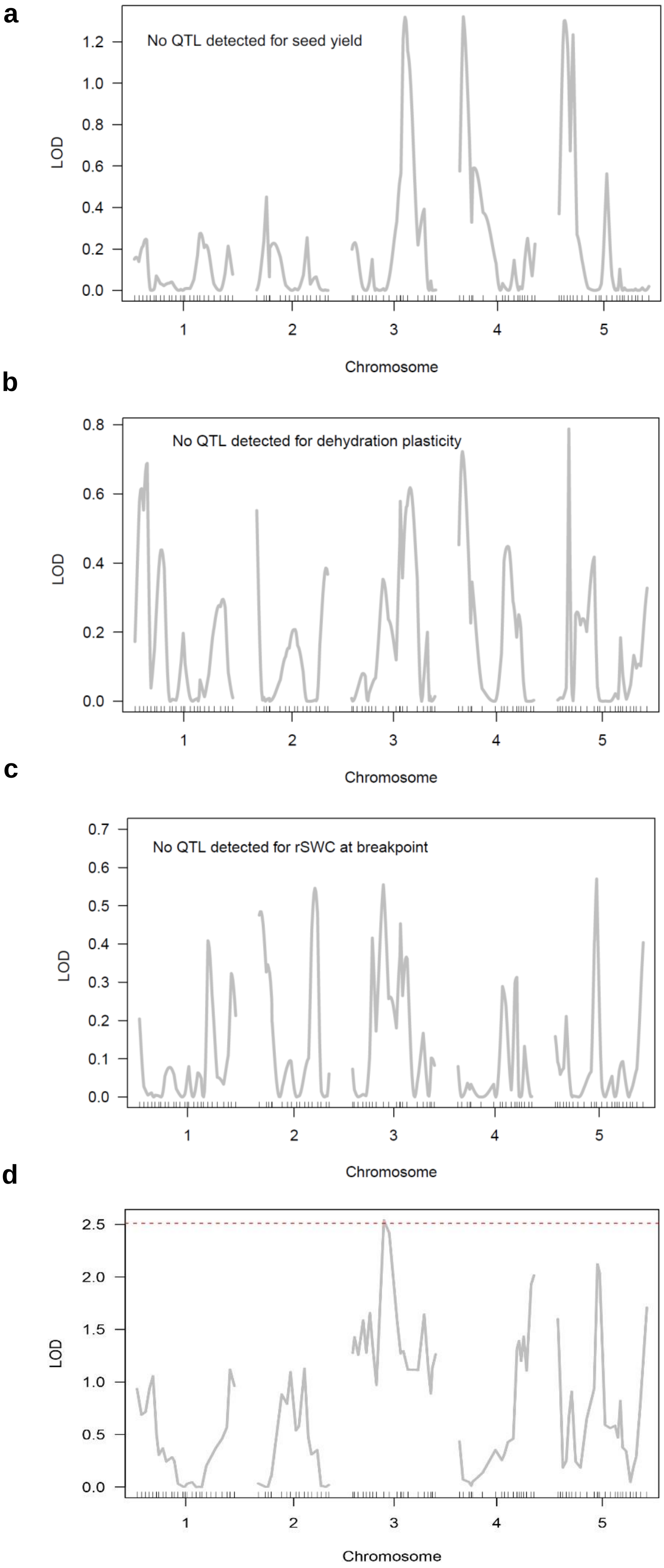

Supplemental Figure 4

Figure S4: Additional QTL mapping results. a - LOD profile for seed biomass, with no significant QTL detected, **b** - LOD profile for dehydration plasticity, with no significant QTL detected, **c** - LOD profile for breakpoint (rSWC), with no significant QTL detected, and **d** – LOD profile for slope 1, with one significant QTL detected. The dashed horizontal red line indicates the 0.05 genome-wide significance threshold

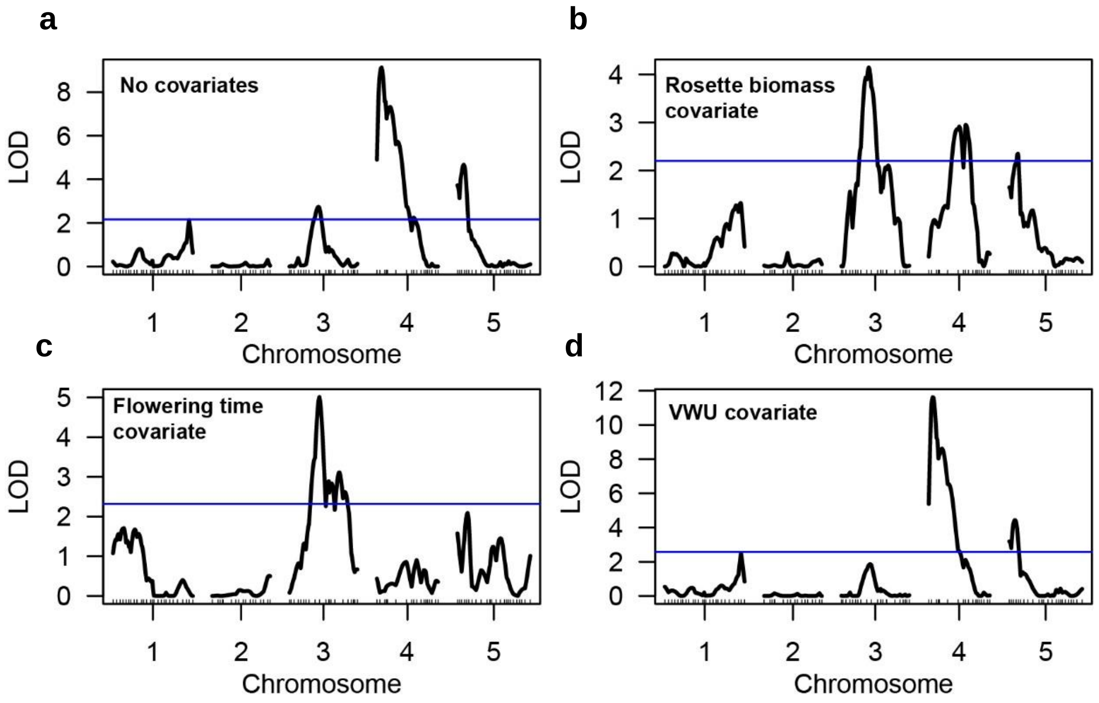

Figure S5: Single QTL mapping for calculated plant water use with and without traits as covariates. a – Without a trait covariate. **b –** With rosette biomass as a trait covariate. **c –** With flowering time as a trait covariate. **d-** With vegetative water use as a covariate.

Supplemental Figure 5

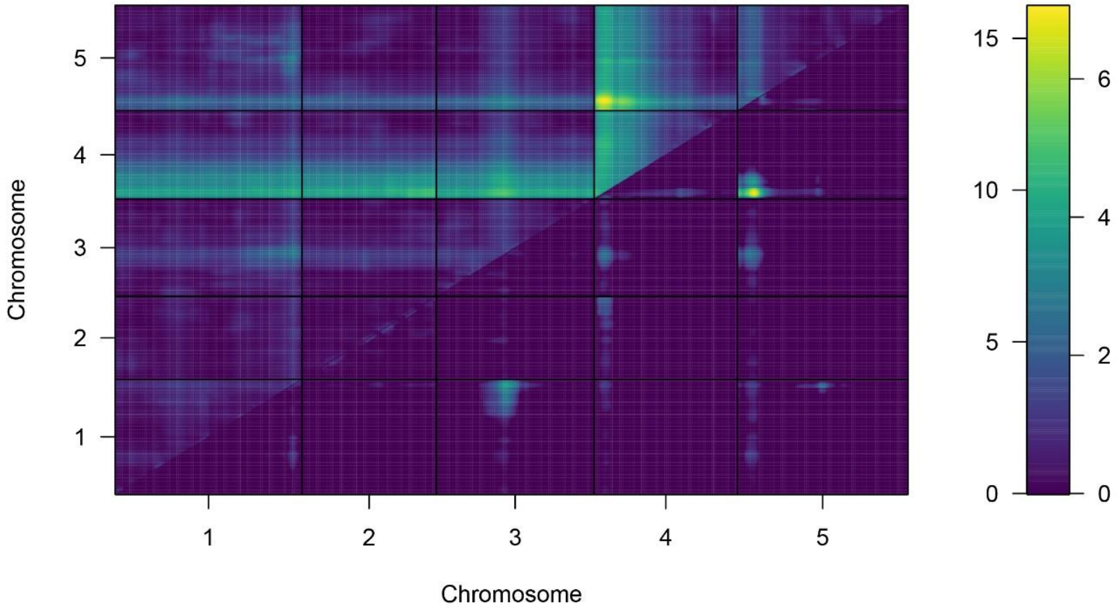

Figure S6: LOD scores for a two dimensional genome scan for calculated plant water use. Values in the upper left triangle represent the full QTL model. Values on the lower right triangle represent the likelihood ratio comparing the full model with QTLs on all chromosomes with the single QTL model, thus indicating the presence of epistatic interactions.

Supplemental Figure 6

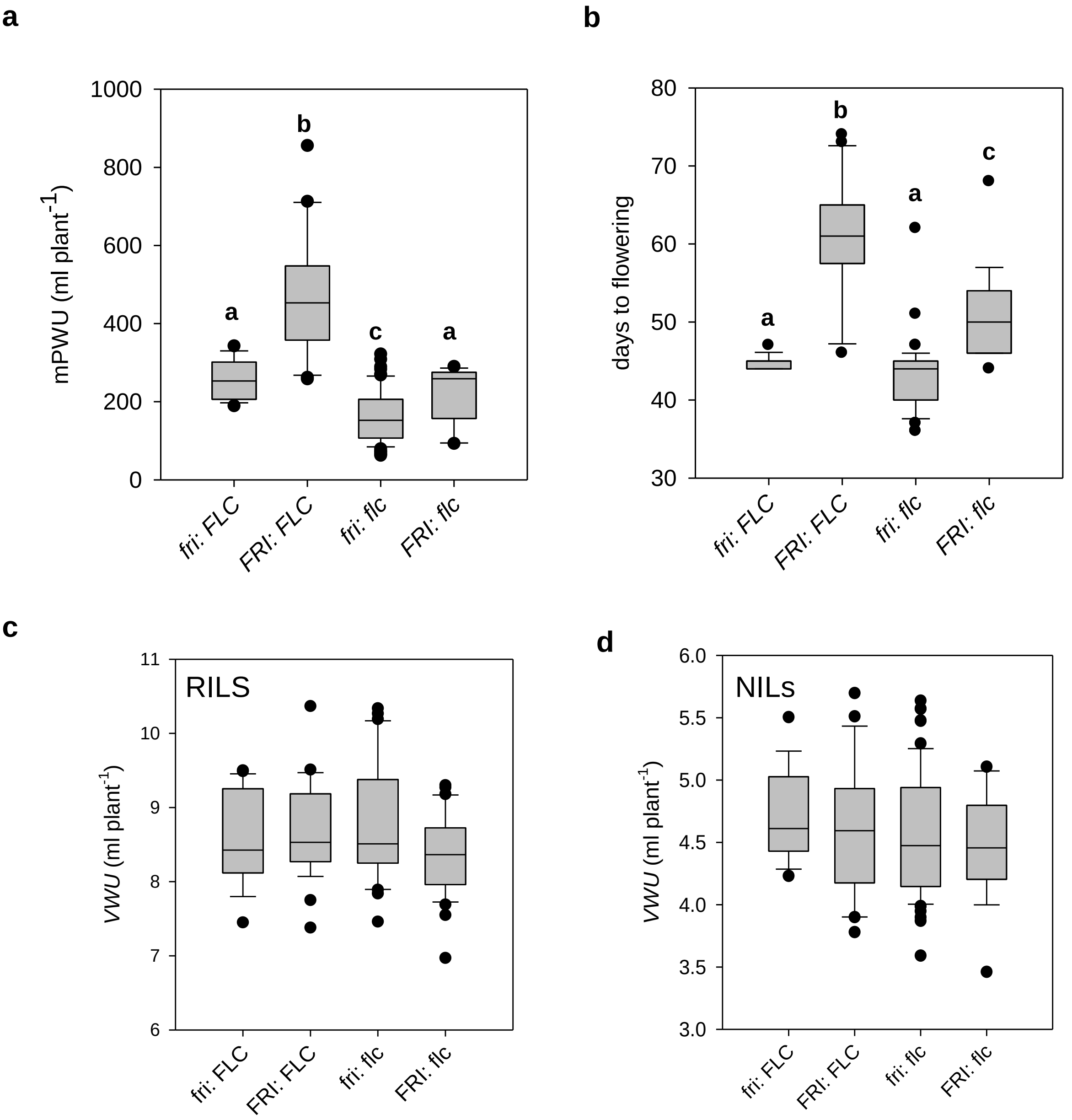

Figure S7: Trait performances of genotypes harbouring different allelic combinations of the *FRIGIDA* (*FRI*) and *FLOWERING LOCUS C* (*FLC*) genes. Boxplots describing the variation for traits assessed for the 4 groups based on allelic combination of *FRI* and *FLC,* **a –** mPWU in the NILs, **b -** days to flowering in the NILs, **c** - VWU based on allelic combinations of *FRI/FLC* in the RILs, and **d** - VWU based on allelic combinations of *FRI/FLC* in the NILs. The letters (a, b, and c) above the boxplot denote the post-hoc Tukey groups, where allelic groups whose letters are different are significantly different from one another for that particular trait at $P < 0.05$. The bold line in the centre of the boxplots represents the median, the box edges represent the 25th (lower) and 75th (upper) percentiles, the whiskers extend to the most extreme data points that are no more than 1.5x the length of the upper or lower segment. Outliers are data points that lie outside the 1.5x interquartile range both above the upper quartile and below the lower quartile.

Supplemental Figure 7

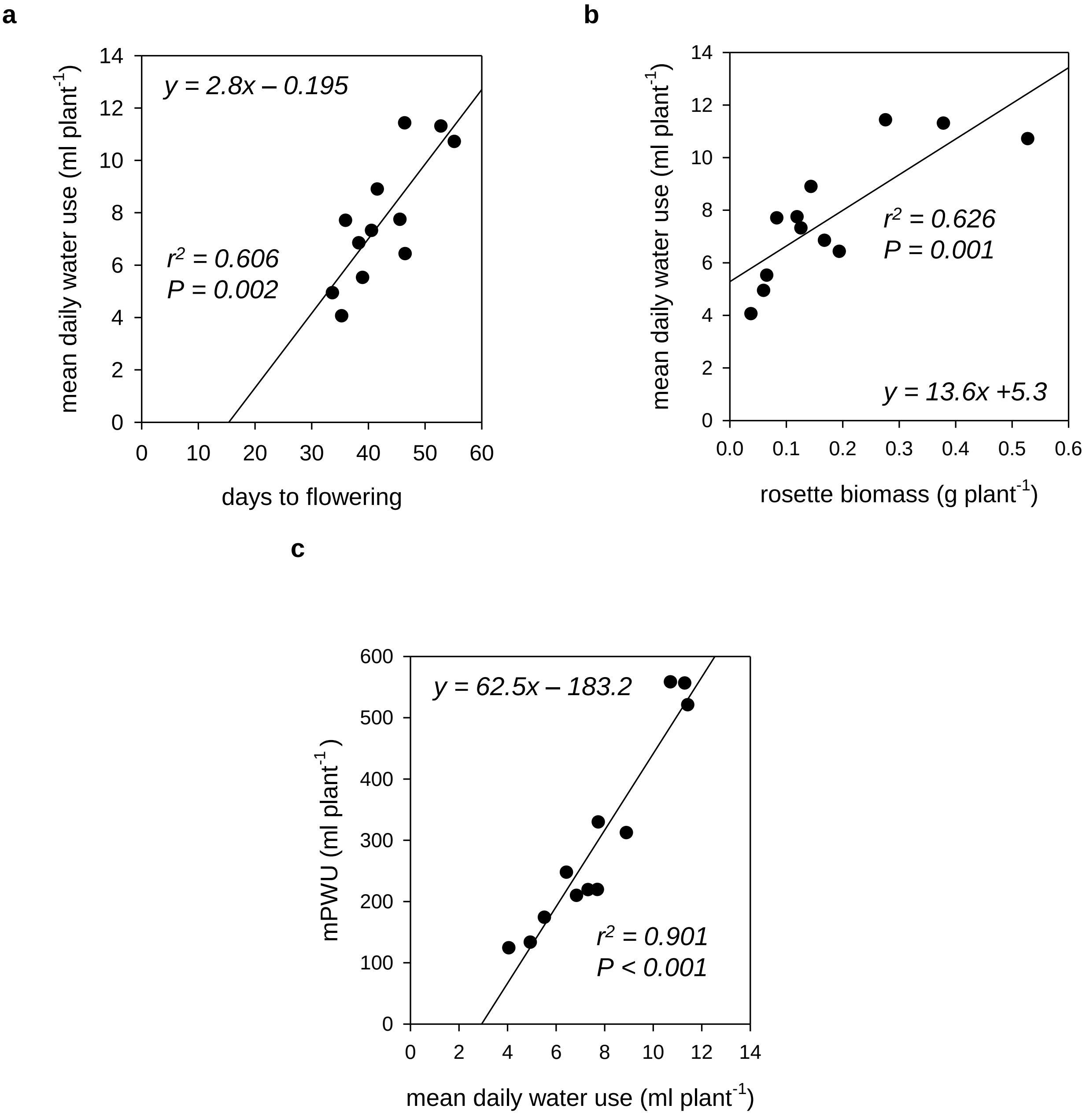

Figure S8 The contribution of mean daily water use in the 12 ecotypes. a - relationship between flowering time and mean daily water use, **b -** relationship between rosette biomass and mean daily water use, and **c -** relationship between mean daily water use and mPWU. The linear model of the relationship between mean long term water use and mean daily water use is provided as the orange fit line. R2 and P values are provided where a significant relationship was identified.

Supplemental Figure 8

a

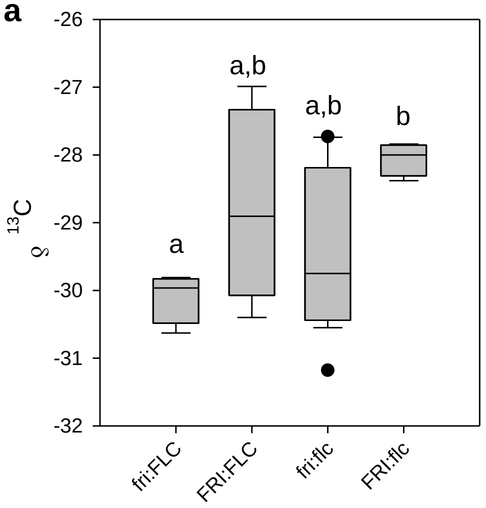

b

FRI/FLC	phenotype	line	Gs (mmol m-2 s-1)	$\delta^{13}C$
FRI: C24, FLC: C24	C24	C24	116.7	-28.1
FRI:C24, FLC:Col	C24 like	M29	118.1	-27.4
FRI:Col, FLC:C24	C24 like	Msub15	121.7	-28.0
FRI:Col, FLC:C24	C24 like	M36	112.0	-28.2
FRI: Col, FLC: Col	Col	Col-0	188.2	-29.9
FRI: Col, FLC:C24	Col like	N13	269.9	-30.1
FRI: Col, FLC:C24	Col-like	N14	274.6	-30.0
FRI:C24, FLC:Col	Col-like	Nsub21	285.5	-30.1
FRI: Col, FLC:C24	Col like	N71	309.2	-30.3

c

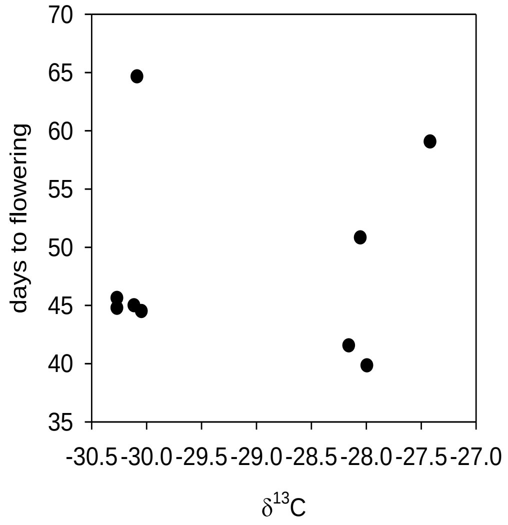

d

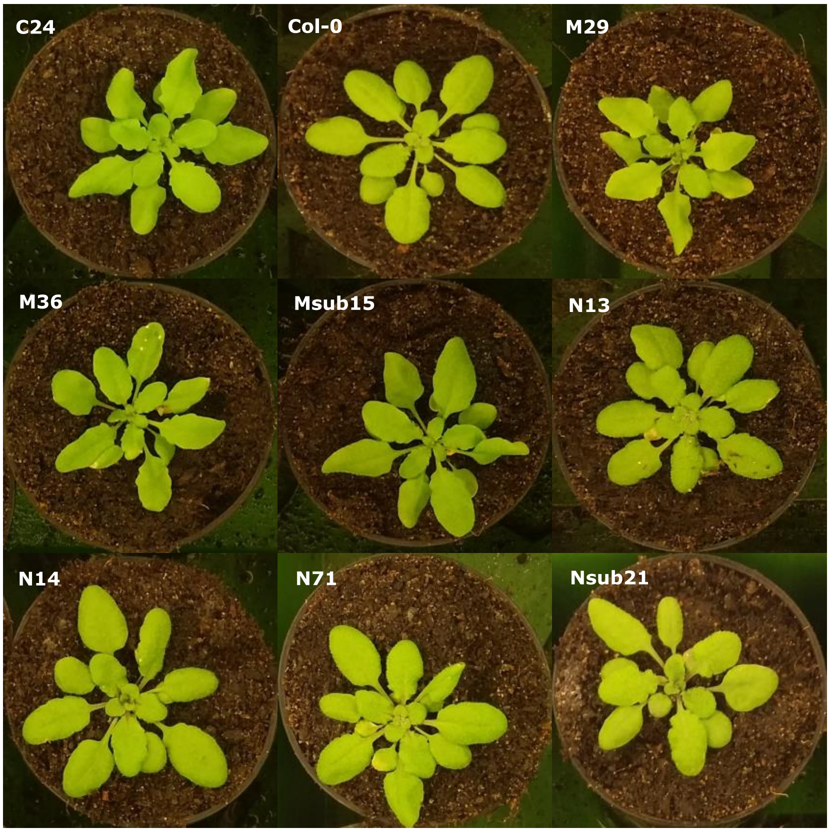

Figure S9: Phenotype of NILs and parental lines. a - boxplots of leaf level WUE ($\delta^{13}C$) for the 4 groups based on allelic combination of both *FRI* and *FLC* in the NILs and both parents. The letters (a, b) denote the post-hoc Games-Howell groups, where allelic groups whose letters are different are significantly different from one another for that trait at $P < 0.05$. The bold line in the centre of the boxplots represents the median, the box edges represent the 25th (lower) and 75th (upper) percentiles, the whiskers extend to the most extreme data points that are no more than 1.5x the length of the upper or lower segment. Outliers are data points that lie outside the 1.5x interquartile range both above the upper quartile and below the lower quartile, **b** - phenotype scoring based on rosette growth (panel C), stomatal conductance (*gs*) and $\delta^{13}C$ measurements. There was a significant negative correlation between gs and $\delta^{13}C$. $r^2 = 0.781$, $P < 0.001$, **c** - relationship between $\delta^{13}C$ and flowering time, and **d** - rosette growth at 25 days post sowing.

a

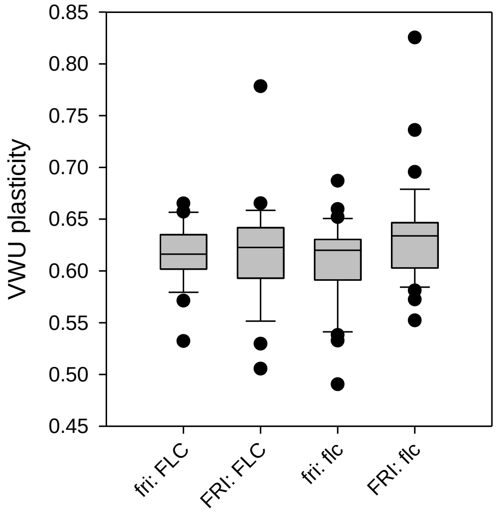

b

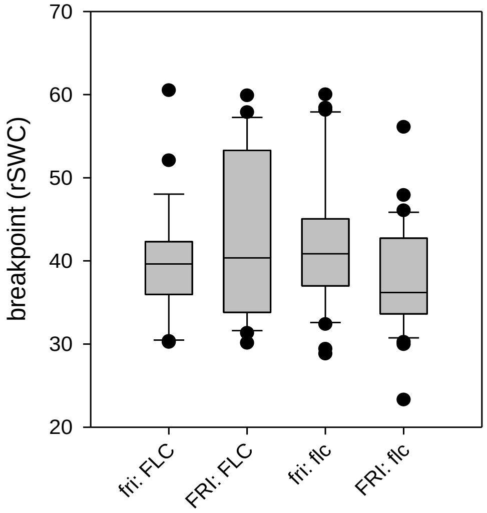

Figure S10: Boxplots of drought response parameters derived from segmented regression analysis based on allelic combinations of FRI/FLC. a - dehydration plasticity (see Table 1), and **b** - breakpoint (rSWC) between segment 1 and 2. Both parameters were calculated using predicted means of the short dehydration experiment performed on the RIL population. No significant differences were detected between the four allelic combinations. The bold line in the centre of the boxplots represents the median, the box edges represent the 25th (lower) and 75th (upper) percentiles, the whiskers extend to the most extreme data points that are no more than 1.5x the length of the upper or lower segment. Outliers are data points that lie outside the 1.5x interquartile range both above the upper quartile and below the lower quartile.

Supplemental Figure 10

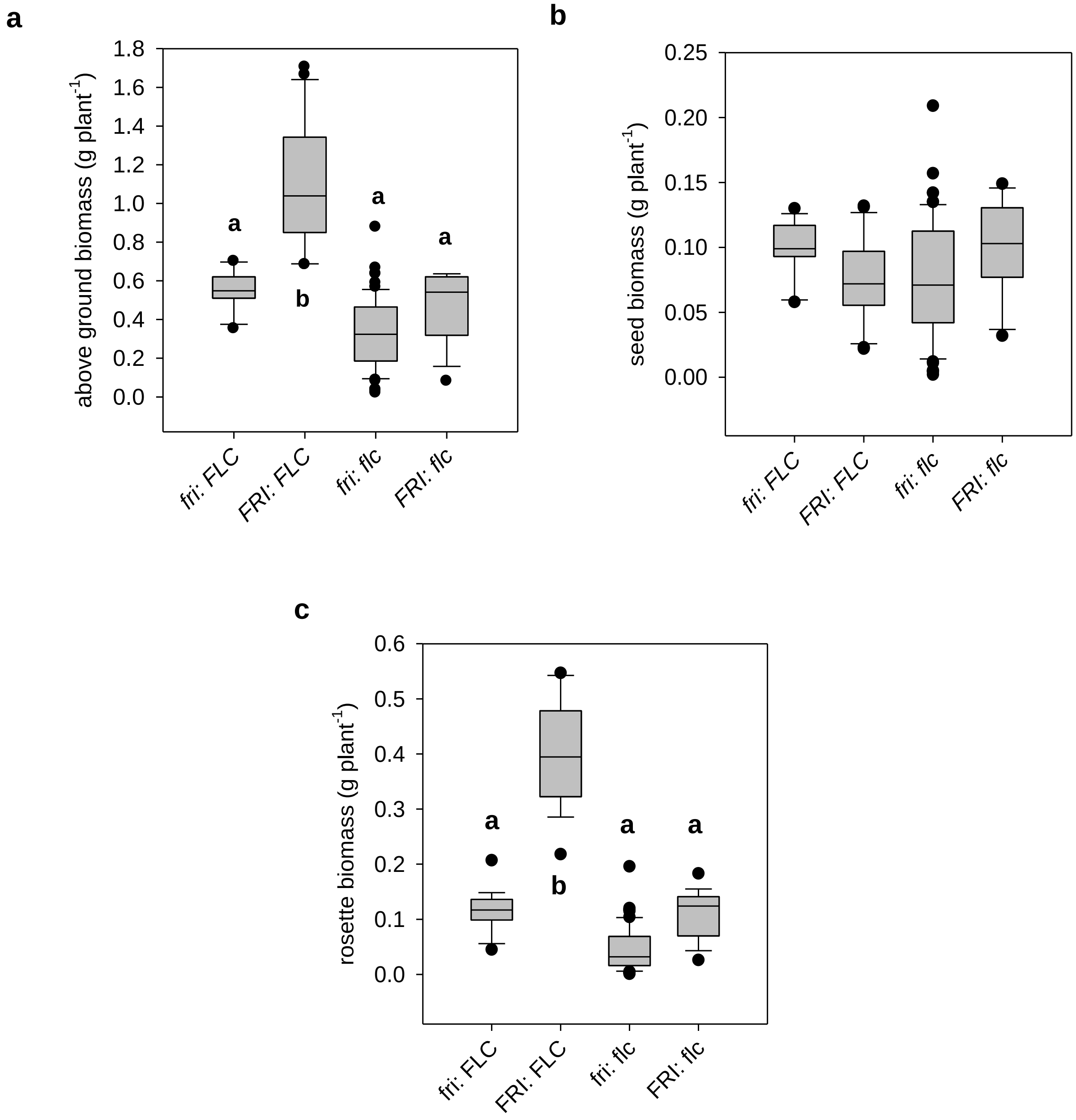

Figure S11: Boxplots of biomass parameters based on allelic combinations of FRI/FLC. In the NILs a – above ground biomass, **b –** seed biomass, and **c –** rosette biomass. The letters (a, b, and c) above the boxplot denote the post-hoc Tukey groups, where allelic groups whose letters are different are significantly different from one another for that trait at $P < 0.05$. The bold line in the centre of the boxplots represents the median, the box edges represent the 25th (lower) and 75th (upper) percentiles, the whiskers extend to the most extreme data points that are no more than 1.5x the length of the upper or lower segment. Outliers are data points that lie outside the 1.5x interquartile range both above the upper quartile and below the lower quartile.

Supplemental Figure 11

a

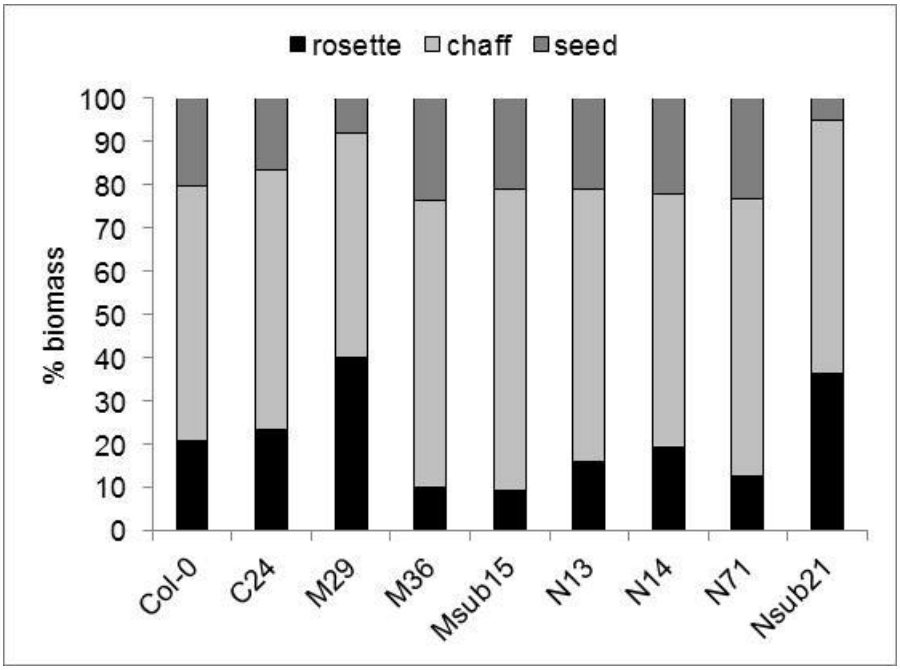

b

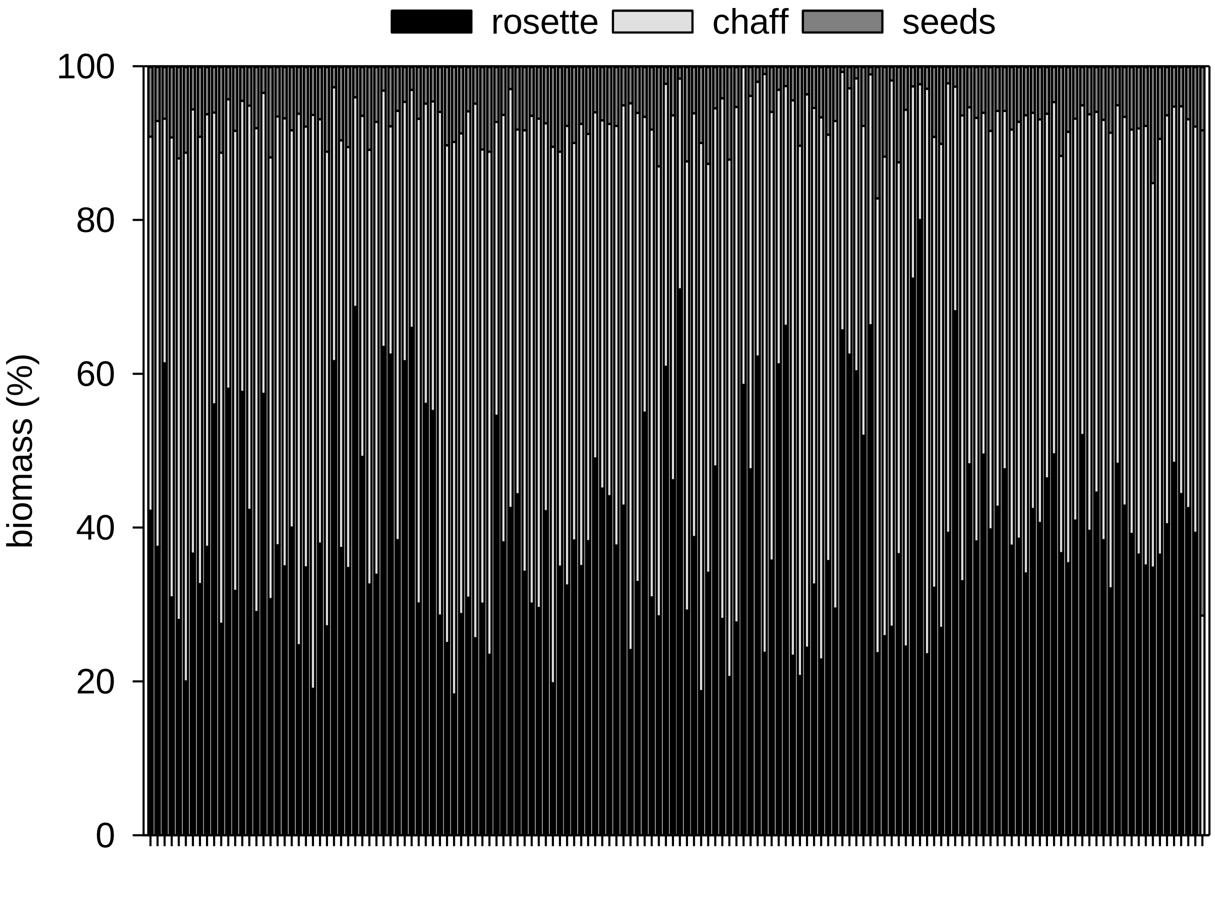

Supplemental Figure 12

Figure S12: Above ground biomass allocation. a - **biomass distribution in the NILs of moderate drought stressed plants, and **b - biomass distribution in 164 RILs including both parents.
